# Oncometabolite D-2HG drives tumor metastasis and protumoral macrophage polarization by targeting FTO/m^6^A/ANGPTL4/integrin axis in triple-negative breast cancer

**DOI:** 10.1186/s13046-025-03282-1

**Published:** 2025-02-06

**Authors:** Siyue Zhang, Ning Zhang, Tong Wan, Yinqiao He, Jie Hao, Yiwei Liu, Yidong Liu, Bing Chen, Wenjing Zhao, Lijuan Wang, Dan Luo, Chao Gao, Qifeng Yang

**Affiliations:** 1 Department of Breast Surgery, General Surgery, Qilu Hospital of Shandong University, No. 107 Wenhuaxi Road, Jinan, Shandong 250012 People’s Republic of China; 2 Biological Resource Center, Qilu Hospital of Shandong University, Jinan, Shandong 250012 People’s Republic of China; 3 State Key Laboratory of Microbial Technology, Shandong University, Qingdao, 266237 People’s Republic of China; 4 Research Institute of Breast Cancer, Shandong University, Jinan, Shandong 250012 People’s Republic of China

**Keywords:** Metastasis, m^6^A methylation, TNBC, D-2HG, ANGPTL4

## Abstract

**Background:**

D-2-hydroxyglutarate (D-2HG), an oncometabolite derived from the tricarboxylic acid cycle. Previous studies have reported the diverse effects of D-2HG in pathophysiological processes, yet its role in breast cancer remains largely unexplored.

**Methods:**

We applied an advanced biosensor approach to detect the D-2HG levels in breast cancer samples. We then investigated the biological functions of D-2HG through multiple in vitro and in vivo assays. A joint MeRIP-seq and RNA-seq strategy was used to identify the target genes regulated by D-2HG-mediated N6-methyladenosine (m^6^A) modification. RNA pull-down assays were further applied to identify the reader that could specifically recognize the m^6^A modification on angiopoietin like 4 (ANGPTL4) mRNA and RNA immunoprecipitation was used to confirm the findings.

**Results:**

We found that D-2HG accumulated in triple-negative breast cancer (TNBC), exerting oncogenic effects both in vitro and in vivo by promoting TNBC cell growth and metastasis. Mechanistically, D-2HG enhanced global m^6^A RNA modifications in TNBC cells, notably upregulating m^6^A modification on *ANGPTL4* mRNA, which was mediated by the inhibition of Fat-mass and obesity-associated protein (FTO), resulting in increased recognition of m^6^A-modified *ANGPTL4* by YTH N6-methyladenosine RNA binding protein F1 (YTHDF1), thereby promoting the enhanced translation of ANGPTL4. As a secretory protein, ANGPTL4 subsequently activated the integrin-mediated JAK2/STAT3 signaling cascade in TNBC cells through autocrine signaling. Notably, the knockdown of *ANGPTL4* or treatment with GLPG1087 (an integrin antagonist) significantly reduced D-2HG-induced proliferation and metastasis in TNBC cells. Additionally, ANGPTL4 was found to promote macrophage M2 polarization within the tumor microenvironment via paracrine signaling, further driving TNBC progression. The association of ANGPTL4 with poor prognosis in TNBC patients underscores its clinical relevance.

**Conclusions:**

Our study unveils a previously unrecognized role for D-2HG-mediated RNA modification in TNBC progression and targeting the D-2HG/FTO/m^6^A/ANGPTL4/integrin axis can serve as a promising therapeutic target for TNBC patients.

**Supplementary Information:**

The online version contains supplementary material available at 10.1186/s13046-025-03282-1.

## Background

Metabolic reprogramming is widely recognized as a fundamental hallmark of cancer [1–3]. Dysregulation of cellular metabolism not only perturbs intracellular signaling pathways but also profoundly reshapes the tumor microenvironment (TME) through the pathological accumulation of metabolites [4]. Among these, fumarate, succinate, and 2-hydroxyglutarate (2HG) have been designated as oncometabolites due to their capacity to disrupt metabolic networks, alter the epigenome, and compromise genomic stability—processes integral to cancer progression [4–6]. 2HG, a by-product of the tricarboxylic acid cycle, has been shown to accumulate aberrantly in various cancers and plays multifaceted roles in both cancer initiation and progression. This metabolite exists in two enantiomeric forms: D-2HG and L-2HG, both of which share structural similarities with α-ketoglutarate (α-KG). Due to this structural similarity, 2HG can competitively inhibit a series of α-KG-dependent dioxygenases, leading to widespread epigenetic modifications, such as histone, DNA, and RNA hypermethylation. Among these epigenetic changes, N6-methyladenosine (m^6^A) methylation stands out as the most prevalent RNA modification in nearly all eukaryotes [7, 8]. This modification exerts a profound influence on mRNA fate by modulating key processes, including alternative splicing, RNA stability, and translation efficiency [9–11]. Extensive research has demonstrated that dysregulated m^6^A modifications of oncogenes or tumor suppressor genes are closely linked to tumorigenesis, cancer progression and drug resistance [12–14].

Breast cancer remains the most prevalent malignancy and the leading cause of cancer-related mortality among women around the globe [15]. The absence of estrogen and progesterone receptors and the lack of ERBB2/HER2 gene amplification or overexpression characterize triple-negative breast cancer (TNBC). Due to TNBC’s highly aggressive nature and the scarcity of effective therapeutic options, even though it makes up only 10–15% of all cases of breast cancer, it is responsible for approximately one-third deaths related to breast cancer [16]. Elevated levels of 2HG, particularly the D-enantiomer, have been observed in breast cancer tissues [17]. Given the context-specific effects of 2HG in cancers, there is an urgent need to further investigate the potential impact of D-2HG on breast cancer. Herein, we first quantified D-2HG levels in both serum and tissue samples from a large cohort of breast cancer patients utilizing an advanced biosensor technique, revealing significant accumulation of D-2HG in TNBC samples. Subsequently, we explored the biological functions of D-2HG in the progression of TNBC. To elucidate the fundamental molecular mechanisms by which D-2HG exerts its oncogenic effects, we explored downstream targets influenced by D-2HG-mediated m^6^A modifications using RNA-seq combined with MeRIP-seq. The integrative sequencing analysis identified angiopoietin like 4 (ANGPTL4) could be significantly regulated by D-2HG in m^6^A modification dependent manner. Moreover, we demonstrated that hypermethylation of *ANGPTL4* mRNA at m^6^A site was recognized by YTH N6-methyladenosine RNA binding protein F1 (YTHDF1), which enhanced the translation of ANGPTL4. Functionally, as secreted protein, we showed that autocrine ANGPTL4 promoted TNBC cell proliferation and metastasis by activating the integrin/JAK2/STAT3 pathway, while paracrine ANGPTL4 triggered protumoral macrophage polarization within the tumor-associated microenvironment, thereby contributing to TNBC progression. Furthermore, we investigated the clinical significance of ANGPTL4 in TNBC, positioning the D-2HG/FTO/m^6^A/ANGPTL4/integrin axis as a promising therapeutic target and prognostic biomarker for patients with TNBC.

## Methods

### Patient samples collection

Serum samples from healthy individuals and patients with breast cancer were collected from Qilu Hospital. All tumor tissues, and adjacent non-cancerous tissues were collected from surgically treated patients diagnosed with breast cancer and stored at − 80 °C until use.

### Patient-derived organoids and treatment

Fresh TNBC tissues were minced into fragments of 1–3 mm^3^ using surgical scalpels on ice, followed by digestion for an hour at 37 °C with gentle shaking in a DMEM/F12 (Macgene)-based digestion medium supplemented with 200 U/mL collagenase I and III (Worthington), 1% BSA (Sigma-Aldrich), 100 μg/mL Primocin (Invivogen), 1 × ITS Supplement (Sigma-Aldrich), 10 mM HEPES, 50 U/mL hyaluronidase (Worthington) and 5 μM Y-27632 dihydrochloride (Sigma-Aldrich). The digested tissues were filtered and centrifuged. The pellets were resuspended and washed once by digestion termination solution (DMEM/F12 supplemented with 0.1% BSA and 100 μg/mL Primocin). To lyse the erythrocytes, the pellets were resuspended and washed once by TAC buffer. Then the cell pellets were washed twice with the digestion termination solution and centrifuged at 300 g for 5 min at 4 °C. Aspirate the supernatant and quickly resuspend the pellet in the growth factor reduced Matrigel on ice. Matrigel containing organoids was seeded on the bottom of pre-heated 48-well culture plate. The culture plate was placed into a humidified incubator at 37 °C and 5% (vol/vol) CO_2_ for 30–60 min to solidify the Matrigel and then overlaid with 300 μl pre-warmed organoid culture medium (bioGenous) to each well. The medium was changed every 2–3 days carefully aspirating medium from the wells and replacing it with fresh, pre-warmed medium. Two weeks later, organoids were passaged with TrypLE (Gibco) for digestion and were re-plated in 384-well screen plates. For determining the effection of D-2HG, the organoids were with different concentration of D-2HG for 5 days, followed by cell viability detection with CellTiter-Glo 3D reagent.

### Cell culture and treatment

Human cell lines, including HEK-293T and various breast cancer cell lines (MCF7, T47D, ZR-75–1, SKBR3, MDA-MB-231, MDA-MB-468, BT549, HCC1937), and MCF-10A (the normal mammary epithelial cell line), were purchased from ATCC. Cell lines were verified by STR Profiling and tested for mycoplasma contamination. HEK-293T, MCF7, MDA-MB-231, MDA-MB-468, BT549 cells were cultured in high glucose DMEM (Macgene). SKBR3 cells were cultured in McCoy's 5A medium (Macgene). T47D, ZR-75-1, and HCC1937 cells were maintained in RPMI-1640 (Macgene). MCF-10A cells were cultured by purchased specific complete medium (Pricella). To isolate mouse primary peritoneal macrophages, peritoneal exudate cells were collected from 4- to 6-week-old C57BL/6 J mice which had been intraperitoneally injected with 3% Brewer’s thioglycolate broth three days earlier and incubated high glucose DMEM (Macgene). Following a 2-h incubation, the medium was changed, and the adherent cells forming the monolayer were utilized as primary peritoneal macrophages for the subsequent experiments. All media contained 10% fetal bovine serum (Gibco), 100 U/ml penicillin (Macgene), and 100 μg/ml streptomycin (Macgene) and cell lines were incubated at 37 ℃ with 5% CO_2_. Cells were treated with D-2HG modified with octyl ester with membrane permeability at indicated concentrations.

### Lentiviral production and infection

To generate *FTO* knockout cell lines, HEK-293T cells were initially transfected with lentiCRISPR V2-sg*FTO* and packaging plasmids (pMD2.G and pxPAX2) using PEI MAX (Polysciences) for lentivirus production. Subsequently, the recombinant lentiviruses were added into cells with 8 μg/ml polybrene (Santa Cruz). Stably expressed single clones were selected by puromycin resistance and validated by western blot.

### Plasmids

*YTHDF1* and *ANGPTL4* expression plasmids were conducted based on pcDNA3.1 vector (Invitrogen). *ANGPTL4* knockdown plasmids were cloned into pLKO.1 vector (Addgene). Point mutations of *YTHDF1* and *ANGPTL4* were constructed using the KOD-Plus-Mutagenesis kit (Toyobo). Sequencing provided confirmation for each construct.

### Plasmid and siRNA transfection

All the plasmids, which include *YTHDF1*-WT, *YTHDF1*-Mut, *ANGPTL4*-WT, *ANGPTL4*-Mut, were transfected into cells by Lipofectamine 2000 (Invitrogen) according to the manufacturers’ instruction. Specific siRNAs of *YTHDF1* and *ANGPTL4* were synthesized by GenePharma Co., Ltd. (Shanghai, China). RFect siRNA/miRNA Transfection Reagent (Bio-trans) were used to transfect siRNA to cells according to the protocol. The sequences are listed in Supplemental Table 1.

### D-2HG measurement

D-2HG measurement was performed using a highly sensitive D-2HG biosensor, which is based on the allosteric transcriptional factor DhdR as described in a previous study [18]. Briefly, serum, tissue supernatant, or cell pellet homogenate (post-centrifugation) were first diluted with a detection buffer containing the purified D-2HG biosensor. The diluted samples were then thoroughly mixed and transferred into a 384-well black plate. After a 10-min incubation at room temperature, the plate was measured using the microplate reader (Perkin Elmer), and the D-2HG concentrations in the samples were calculated using the emission ratio according to the normalized dose–response curve.

### RNA isolation and qPCR

PrimeScript™ FAST RT reagent Kit (Takara) was applied to reverse transcribe RNA into cDNA after it had been extracted using RNAiso Plus reagent (Takara). The qPCR SYBR Green Mix (YEASEN) was then used, in accordance with the manufacturer's instructions, for quantitative PCR (qPCR) on a LightCycler® 480 real-time PCR system (Roche). The relative mRNA abundance was normalized to the reference gene *ACTIN*, and calculations were performed using the comparative Ct (− ΔCt) method. Primer sequences are provided in Supplemental Table 1.

### Protein extraction and western blot

Transfected or treated cells were subjected to protein extraction, followed by lysis using RIPA buffer (Beyotime) containing the cocktail of protease and phosphatase inhibitors (Roche). The BCA protein assay kit (Millipore) was utilized to ascertain protein concentrations. Protein samples ranging from 10 to 30 μg were separated by 8% or 10% SDS-PAGE and transferred onto methanol-activated 0.22-μm PVDF membranes (Millipore). The membranes were blocked using 5% non-fat milk, and then subjected to an overnight incubation using certain primary antibodies, succeeded by matching secondary antibodies (Cell Signaling Technology). Detection was carried out using the ECL detection system (TECAN), with ACTIN and GAPDH serving as loading controls. 

### ELISA

Commercially available ELISA kits (Lianke Biotech) were used to quantify the amount of ANGPTL4 in serums and cell supernatants. The absorbance was recorded at 450 nm with a Perkin Elmer microplate reader. The concentrations of ANGPTL4 were computed using the standard curve as a guide.

### H&E staining and immunohistochemistry

For H&E staining, paraffin-embedded lung tissues were sectioned at 5 µm thickness, deparaffinized in xylene, and rehydrated using a series of gradient ethanol. Slides stained with hematoxylin and eosin were mounted with coverslips and photographed under a Leica light microscope.

For IHC staining, the procedure was performed according to the protocol of M&R HRP/DAB detection IHC kit (Vazyme). Briefly, sections were deparaffinized as described, and then antigen was retrieved using Tris–EDTA buffer (pH 9.0). The sections treated with hydrogen peroxide blocking reagent were subsequently incubated with primary antibodies at 4 °C overnight. The following day, the sections were washed with PBS and treated with HRP polymer buffer at room temperature for 20 min, stained with diaminobenzidine, counterstained with hematoxylin, dehydrated, mounted with neutral resin, and observed by an Olympus microscope.

### Cell proliferation assays

For the 3-(4,5-dimethylthiazol-2-yl)-2,5-diphenyltetrazolium bromide (MTT) assay, 1.5 × 10^3^ cells per well were cultured in 96‐well plates and subjected to the treatments specified in the figure legends. Each well received 20 µL of freshly made MTT solution (5 mg/mL in PBS) at the designated time periods, and the wells were incubated for 4 h. Afterward, each well received 100 µL of dimethyl sulfoxide (DMSO). The plates were gently shaken, and absorbance was measured at 450 nm using a microplate reader (Perkin Elmer).

For the 5‐ethynyl‐2’‐deoxyuridine (EdU) assay, 1 × 10^4^ cells per well were seeded in 96‐well plates. After being labeled with EdU solution, cells were cultured for 2 h at 37 °C. The cells were then fixed with 4% paraformaldehyde, permeabilized using 0.5% Triton X-100, stained with Apollo staining mixture, counterstained by Hoechst solution, and examined using a fluorescence microscope (ZEISS).

For the colony formation assay, 8 × 10^2^ cells per well were cultured in 6-well plates. After overnight incubation, the cells were exposed to a range of D-2HG concentrations and left to incubate for a further two to three weeks. After 20 min of 100% methanol fixation, the colonies stained for 30 min with 0.75% crystal violet, and colonies containing over 50 cells were counted.

### Transwell assays

To perform the migration assay, 6 × 10^4^ cells were transferred to the upper chambers (Corning) in 300 µL of starvation media, while 700 µL of culture medium with 20% FBS was added to the lower chamber. The migrated cells were proceeded by methanol fixation and crystal violet staining, and then were quantified under a microscope after 24–48 h. To perform the invasion assay, Matrigel (Corning) pre-coated chambers were utilized, and the following procedures can be referred to the migration assay.

### Wound healing assay

2.5 × 10^5^ cell were seeded in a 12‐well plate. Once the confluent monolayer cells formed, a straight line was scraped across the monolayer using a 10 µL pipette tip. A phase-contrast microscope was used to observe the wound area at 0 and 24 h.

### Mammosphere formation assay

6 × 10^2^ cells were seeded into 96-well ultra-low attachment plates (Corning). The plates were incubated at 37 °C with 5% CO_2_ for two weeks until mammospheres became visible. The mammospheres were then photographed and counted using a microscope.

### RNA-seq analyses

Total RNAs were extracted using RNAiso Plus reagent (Takara). RNA quality assessment, library preparation and sequencing were carried out at Novogene, Inc. (Tianjin, China). Raw reads were filtered using Trim Galore (v0.6.10) [19] and aligned to the hg38 genome with HISAT2 (v2.2.1) [20]. FeatureCounts (v2.0.6) [21] was employed to quantify reads based on the annotation file downloaded from the GENCODE database (v32, GTF format for human). Genes expressed in fewer than 75% of the total samples were removed from the expression matrix. Differentially expressed genes between D-2HG and DMSO groups were identified using DESeq2 (v1.40.2) [22] with thresholds of log2FoldChange > 1 and p < 0.05. ClusterProfiler (v4.8.2) [23] was applied for functional enrichment analysis.

### Global RNA N6-methyladenosine analysis

The EpiQuik m^6^A RNA Methylation Kit (Epigentek) was used to assay the amount of m^6^A in total RNA. Briefly, 200 ng of total RNA and the binding solution were added to the wells for the experiment. The capture antibody solution, detection antibody solution, enhancer solution, developer solution, and stop solution were applied to each well in order specified procedures. Using a Perkin Elmer microplate reader, the relative m^6^A levels were calculated according to the absorbance at 450 nm.

### m^6^A dot blot

To assess the global abundance of m^6^A modification, a dot blot assay was performed. Using the Dynabeads mRNA Purification Kit (Invitrogen), poly(A) + mRNA samples were purified and then denatured and spotted onto a nylon membrane (Amersham, GE Healthcare). The membrane was air-dried, cross-linked at 254 nm (SCIENTZ), and blocked with 5% non-fat milk. After overnight incubation with m^6^A antibody (Synaptic Systems), the membrane was washed with PBST and incubated with an HRP-conjugated anti-mouse antibody (Cell Signaling Technology), followed by exposure to ECL (Yeason) using a visualizer (Tanon). The membrane dyed with 0.2% methylene blue served as the loading control and was imaged accordingly.

### N6-methyladinosine modification prediction

SRAMP (http://www.cuilab.cn/sramp) is a robust m^6^A site prediction tool that utilizes sequence-derived features and a machine learning framework [24]. Potential m^6^A sites in the human *ANGPTL4* transcript were predicted using the full transcript mode, with the results listed in Supplementary Table 4.

### RNA pull-down

Biotin-labeled single-stranded RNA (ssRNA) containing either methylated or unmethylated adenosine was synthesized by GenScript Biotech Corporation (Nanjing, China), with sequences provided in Supplementary Table 1. Briefly, 0.1 nmol of biotin-labeled ssRNA was incubated with 0.5 mg of magnetic beads (Vazyme) in binding buffer for 30 min at room temperature. The RNA-bound beads were incubated with 200 μg of MDA-MB-468 cell lysate overnight at 4 °C. The RNA-binding proteins were eluted with 1 × SDS buffer and analyzed by silver staining (Beyotime) or western blot. The m^6^A-binding proteins in the gel slices were sent to Lianchuan Bio (Hangzhou, China) for mass spectrometry analysis.

### RNA–protein immunoprecipitation (RIP)–qPCR

The Magna RIP Kit (Millipore) was used to conduct the RIP assay to investigate the direct interaction between YTHDF1 and the *ANGPTL4* transcript. In RIP lysis buffer, which also included RNase inhibitors and a cocktail of protease inhibitors, cells were lysed. After being coated with certain antibodies or IgG, Protein A/G magnetic beads were added into the cell lysate. The bead-antibody complex was rotated at 4 °C overnight. Protease K was used to digest the beads following washing with RIP washing buffer. The RNA from both the input and immunoprecipitated samples were isolated using 100% ethanol and phenol/chloroform/isoamyl alcohol (125:24:1). The RNA was then quantitatively analyzed by qPCR and agarose gel electrophoresis.

### Methylated RNA immunoprecipitation (MeRIP)-seq and MeRIP-qPCR

The MeRIP assay was carried out using the riboMeRIP m^6^A Transcriptome Profiling Kit (Ribobio) with minor modifications. 300 µg of total RNA was purified with 3 M sodium acetate (pH 5.2) and 100% ethanol after being chemically fragmented into 100–150 nucleotide lengths using fragmentation buffer of which 1/10 was used as input and the remainder was incubated with magnetic beads conjugated with the anti-m^6^A antibody for 2 h at 4 °C. The methylated RNA was then eluted over 1 h at 4 °C and subsequently purified with 3 M sodium acetate (pH 5.2) and 100% ethanol. The immunoprecipitated RNA and input from each sample were used for further MeRIP-seq or qPCR analysis.

For MeRIP-seq, an Qsep100 Analyzer (BiOptic) was used to determine the RNA fragments and quality of both IP and rRNA depleted input samples (Ribo-MagOff rRNA Depletion Kit, Vazyme) prior to RNA library preparation using VAHTS Universal RNA-seq Library Prep Kit (Vazyme) and sequenced by Novogene (Tianjin, China). Reads from MeRIP-seq were first trimmed using Trim Galore (v0.6.10) [19] to remove adapters and low-quality sequences. Using Bowtie2 (v2.5.4) [25], the trimmed reads were then aligned to rRNA sequences (downloaded from the National Center for Biotechnology Information Nucleotide database) to retain unmapped read. HISAT2 (v2.2.1) [20] was then used to map the remaining reads to the human genome (hg38). To identify robust differential peaks between D-2HG and DMSO groups, fragment lengths of the BAM files were predicted using MACS2 (v2.2.8) [26], and these were subsequently used in MACS2 callpeak with the –nomodel option. After peak calling, Deeptools (v3.5.5) [27] and MACS2 bdgdiff were used together to identify differential peaks (log10(LR) > 4 and log2FoldChange > 2) between D-2HG and DMSO groups, which can be then annotated to protein coding genes. The m^6^A-enriched motifs in each group were identified using HOMER (v4.1.1) [28]. Additionally, the distribution of peaks from D-2HG and DMSO across various functional regions was determined using the Guitar R package (v2.16.0) [29]. The proportion of peaks in different functional regions was then visualized using ChIPseeker (v1.36.0) [30]. Finally, the results from RNA-seq and MeRIP-seq were integrated by identifying common genes for further analysis.

For the MeRIP-qPCR analysis, primers were designed based on the specific m^6^A modification sites predicted with “very high confidence” using the SRAMP. The relative m^6^A enrichment was calculated referring to the expression of the input RNA. Supplementary Table 1 contains a list of primer sequences that were employed.

### MeCLIP-seq analysis

MeCLIP-seq fastq files were obtained from the GEO database (accession number GSE147440). The MeCLIP-seq analysis pipeline was executed using a Snakemake workflow developed by Justin et al. with default parameters [31]. The resulting BAM files were then converted to BigWig format using Deeptools bamCoverage with the options “–binSize 1 –normalizeUsing RPGC”. The m^6^A binding motif in *ANGPTL4*, along with both IP and input results from MeRIP-seq and MeCLIP-seq were visualized using the Integrative Genomics Viewer (v2.16.1).

### mRNA stability assay

Actinomycin D (ActD, AAT Bioquest), a transcriptional inhibitor, was used to treat the cells for 0, 3, and 6 h. qPCR assay was used to measure the amounts of *ANGPTL4* mRNA. The data were calculated using the comparative Ct (− ΔCt) method.

### Polysome profiling assay

Confluent cells, transiently transfected with siNC or si*YTHDF1*, were cultured in 10 cm dish. Prior to being collected, cells were exposed to a 10-min treatment of 100 μg/mL cycloheximide (CHX, Sigma-Aldrich). Following lysing the cells on ice, the lysate was centrifuged. Onto a gradient of 10/50% w/v sucrose, the supernatant was loaded, followed by centrifugation at 4 °C for 3 h at 36,000 rpm using a Beckman SW41 Ti rotor. After that, the sample was divided into fractions and examined using a fraction collector (FC203B, Gilson) and a Gradient Station (BioCamp) coupled with an ECONOUV monitor (BioRad). Total RNA from each fraction was isolated using RNAiso Plus reagent (Takara) for subsequent qPCR analysis of the *ANGPTL4* transcript.

### Flow cytometry

Tumors were isolated from mice, minced, and incubated for one hour at 37 °C in RPMI-1640 media with 1 mg/mL collagenase I (Worthington) and 0.2 mg/mL DNase I (Sigma-Aldrich). Single-cell suspensions were obtained after filtration using cell stainers (70 μm, Corning). Single-cell suspensions were incubated with Fc receptor blocking buffer (Anti-Mouse CD16/32 Antibody, Elabscience) for 15 min at 4 °C, followed by labeling on ice for 60 min with anti-CD86 (Elabscience), anti-CD206 APC (Elabscience), and anti-F4/80 FITC (Elabscience). The samples were washed, resuspended in FACS buffer (2% FBS in PBS), and analyzed on a flow cytometer. FlowJo software was used to analyze the data.

### Immunofluorescence

Cells seeded on glass cover slides were fixed with 4% paraformaldehyde followed by permeabilization using 0.4% Triton X‐100. After one-hour 5% BSA blocking, the cells were incubated with specific primary antibodies overnight at 4 °C, and subsequently stained with Alexa Fluor-labeled secondary antibodies (ZSBG-BIO) for 2 h at room temperature in the dark. The ProLong Diamond reagent (Invitrogen) was used to mount the slides. Fluorescence photos were captured and processed by fluorescence microscope (ZEISS).

### In vivo tumorigenesis and metastasis assays

Six-weeks female BALB/c nude mice were acquired from Beijing Vital River Laboratory Animal Technology Co., Ltd. (Beijing, China). Mice were housed in specific pathogen-free (SPF) animal facilities at the Laboratory Animal Center of Qilu Hospital, Shandong University.

Subcutaneous injection was performed on thigh of nude mice using 5 × 10^6^ MDA-MB-231 or 5 × 10^5^ 4T1-Luc cells that had been resuspended in 150 μl PBS. After the average size of the subcutaneous tumors was 50 mm^3^, the mice were split into two equal subgroups at random. One subgroup received intraperitoneal injections of D-2HG at a dosage of 250 mg/kg for the indicated duration, while the other subgroup received an equal volume of vehicle control solution.

For the in vivo metastasis assay, intravenous injections were performed into the tail vein using either 1 × 10^5^ 4T1 cells or 5 × 10^5^ MDA-MB-231 cells. After three weeks, all mice were randomly divided into two equal subgroups. One subgroup was treated with intraperitoneal injections of D-2HG at a dosage of 250 mg/kg every three days for the indicated duration, while the other subgroup received an equal volume of vehicle control solution. After forty days, metastasis was monitored using IVIS imaging. After the mice were sacrificed, their lungs were taken out for further examination.

### Statistical analysis

Student's t-test was utilized for comparing two groups, while one-way ANOVA was employed for comparing multiple groups. Statistical analyses were carried out using GraphPad Prism 9 and OriginPro 2023 software. Data were expressed as mean ± SD, with *p* < 0.05 indicating statistical significance.

## Results

### D-2HG accumulates in triple-negative breast cancer

**Fig. 1:**
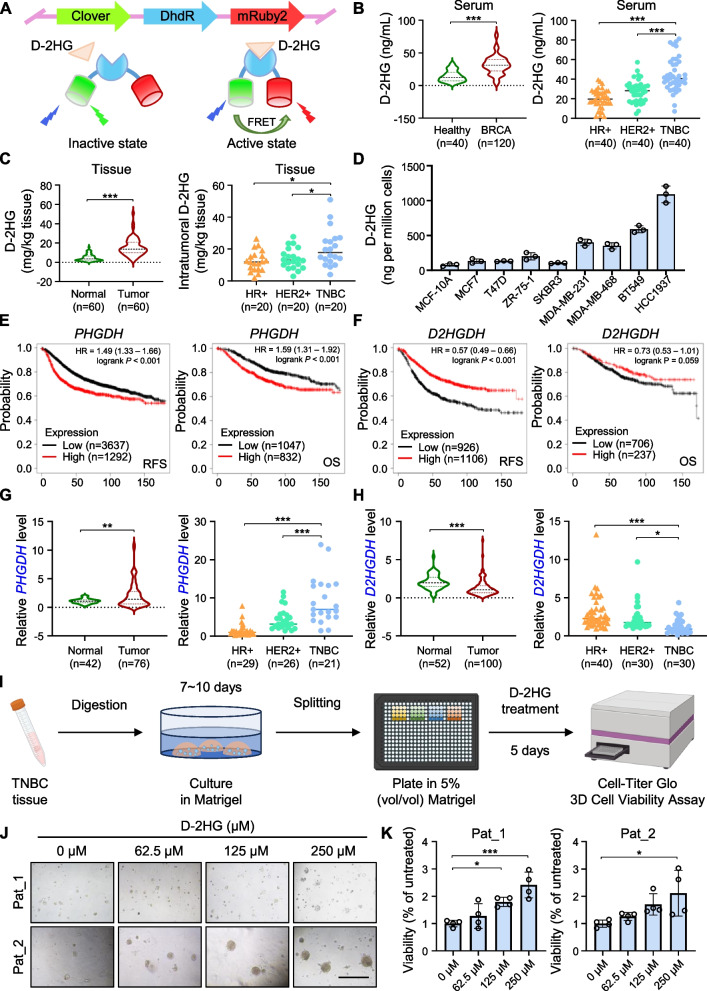
D-2HG prominently accumulates in TNBC and promotes the proliferation of TNBC organoids. **A** Schematic diagram of D-2HG biosensor in the absence or presence of D-2HG. **B** Violin plot (left) and scatter plot (right) of D-2HG levels in serum of healthy controls and breast cancer (BRCA) patients measured by the D-2HG biosensor. **C** Violin plot (left) and scatter plot (right) of D-2HG levels in paired BRCA tumor tissues and normal tissues measured by the D-2HG biosensor. **D** D-2HG levels measured in BRCA cell lines. **E** Recurrence-free survival (RFS; left) and overall survival (OS; right) in BRCA patients with high or low levels of *PHGDH* from Kaplan–Meier plotter database. **F** RFS (left) and OS (right) in BRCA patients with high or low levels of *D2HGDH* from Kaplan–Meier plotter database. **G** Violin plot (left) and scatter plot (right) of *PHGDH* mRNA levels in BRCA tumor tissues versus normal tissues from the Qilu cohort. **H** Violin plot (left) and scatter plot (right) of *D2HGDH* mRNA levels in BRCA tumor tissues versus normal tissues from the Qilu cohort. **I** Schematic overview of organoid isolation, culture and application in viability assay. **J** Representative brightfield images of organoids derived from two different TNBC patients after D-2HG treatment for 5 days. Scale bar: 200 μm. **K** Statistical analysis of organoid viability assay following D-2HG treatment for 5 days. *P < 0.05, **P < 0.01, ***P < 0.001

To investigate whether D-2HG accumulates in breast cancer, we initially employed a biosensor with high accuracy and precision to measure D-2HG levels in serum and tissue homogenate samples from breast cancer patients [18]. The biosensor is based on Förster resonance energy transfer (FRET) and utilizes DhdR as the D-2HG specific biorecognition element inserted between two fluorescent proteins, Clover and mRuby2 (Fig. 1A). According to our findings, breast cancer patients' serum levels of D-2HG were higher than that in healthy individuals, and the levels in paired tumor tissues were also higher than in adjacent non-tumor tissues. Moreover, D-2HG showed the highest accumulation in the TNBC subtype in both serum and tissue samples (Fig. 1B-C). Similarly, TNBC cell lines exhibited significantly higher D-2HG levels (Fig. 1D). We further investigated the reasons for D-2HG accumulation inside the tumors. The cBioportal database analysis indicated that the majority of breast cancer patients do not harbor mutations in isocitrate dehydrogenase 1 and 2 (IDH1 and 2) and the only one case that harbors is HR + sample (Figure S1A), indicating the prevalent accumulation of D-2HG in TNBC samples was not due to *IDH1/2* mutations as the common case in leukemia and glioma. We noticed that some patients exhibited phosphoglycerate dehydrogenase (PHGDH) amplification, which can promote D-2HG accumulation [32, 33], or D-2-hydroxyglutarate dehydrogenase (D2HGDH) deletion, which prevents D-2HG accumulation [34] (Figure S1B). Further correlation analysis of gene alterations and expression confirmed that patients carrying *PHGDH* amplification showed higher expression levels, whereas those with *D2HGDH* deletion had lower expression levels (Figure S1C-D). Moreover, Kaplan–Meier plotter database analysis suggested that high *PHGDH* expression and low *D2HGDH* expression are both strongly connected with a poor outcome for breast cancer (Fig. 1E-F). The qPCR analysis of *PHGDH* and *D2HGDH* expression in tissue samples, along with TCGA database analysis, revealed that *PHGDH* expression was highest and *D2HGDH* expression was lowest in TNBC tumors compared to other breast cancer subtypes (Fig. 1G-H, Figure S1E-F). Comparable results were noted in cell lines of breast cancer (Figure S1G-H), indicating D-2HG accumulation was prominently regulated by PHGDH and D2HGDH in TNBC. Considering the results above, we primarily focused on the role of D-2HG in TNBC.

### D-2HG promotes the proliferation and metastasis of TNBC in vitro and in vivo

**Fig. 2:**
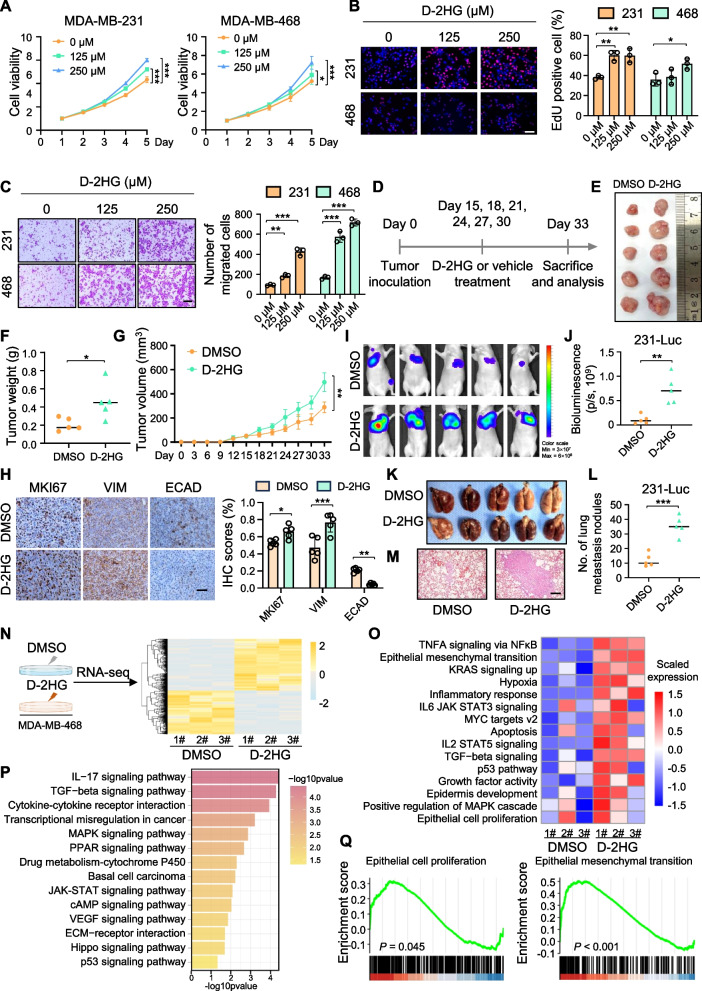
D-2HG promotes the proliferation and metastasis of TNBC cells in vitro and in vivo. **A** MTT assay assessing the effects of D-2HG on TNBC cell proliferation and viability. **B** Representative images and quantification of the EdU assay in TNBC cells. Scale bar: 100 μm. **C** Representative images and quantification of the migration assay in TNBC cells. Scale bar: 200 μm. **D-G** Tumor growth analysis following subcutaneous injection of MDA-MB-231 cells with or without D-2HG treatment (*n* = 5/group). Schematic strategy (D) for D-2HG or vehicle administration in the mouse model. Tumor size (E), tumor weight (F) and tumor growth curve (G) were compared and analyzed. **H** Representative images (left) and quantification (right) of MKI67, VIM and ECAD immunohistochemical staining of xenograft sections. Scale bar: 100 μm. **I-L** Tumor metastasis analysis following tail vein injection of MDA-MB-231-Luc cells with or without D-2HG treatment (*n* = 5/group). Bioluminescent imaging (BLI) images (I) and quantification (J) of BALB/c nude mice after treatment. Images (K) and quantification (L) of lung metastatic nodules from BALB/c nude mice at the endpoint. **M** Representative images of H&E staining of lung tissues from BALB/c nude mice injected with D-2HG or vehicle. **N** Heatmap of significantly differentially expressed genes in MDA-MB-468 cells treated with D-2HG or DMSO for 48 h (3 replicates/group). **O** GSVA analysis of differentially expressed pathways. **P** Gene ontology analysis of differentially expressed genes. **Q** GSEA analysis of RNA-seq results demonstrating enrichment of epithelial cell proliferation and epithelial mesenchymal transition pathways in D-2HG-treated groups. *P < 0.05, **P < 0.01, ***P < 0.001

To explore whether D-2HG exerts an oncogenic effect in vitro, we first developed patient-derived organoids from TNBC tumors (Fig. 1I). The viability of TNBC organoids gradually increased with the administration of rising concentrations of D-2HG (Fig. 1J-K). Next, we performed phenotypic experiments on TNBC cells treated with gradient concentrations of D-2HG. MTT, EdU staining, colony formation, and flow cytometry assays showed enhanced cell proliferation and cell cycle progression following D-2HG treatment (Fig. 2A-B, Figure S2A-B). Additionally, we observed that D-2HG-treated MDA-MB-468 cells exhibited a mesenchymal phenotype, characterized by loss of intercellular adhesion and spindle-like morphology (Figure S2C). Moreover, D-2HG enhanced the migratory and invasive capabilities of MDA-MB-231 and MDA-MB-468 cells (Fig. 2C, Figure S2D-E). Furthermore, D-2HG treatment also led to increased mammosphere formation derived from TNBC cells (Figure S2F). Immunofluorescence assay showed that D-2HG-treated TNBC cells displayed decreased E-cadherin (ECAD) signals (Figure S2G). These findings indicated that D-2HG could promote the proliferation, migration, and invasion of TNBC cells.

Further, we employed tumorigenesis and lung metastasis models to validate the D-2HG’s tumor-promoting effects in vivo. MDA-MB-231 cells were implanted into the flanks of nude mice by subcutaneous injection. After tumor formation, two groups of nude mice were randomly assigned, and each group received treatment with either DMSO or D-2HG every three days (Fig. 2D). Significantly higher tumor weight and growth rate were observed in the D-2HG group as compared to mice treated with DMSO (Fig. 2E-G). The immunohistochemistry assay was performed to evaluate the proliferative and metastatic levels in the xenograft tumors. The tumors treated with D-2HG exhibited increased Marker of proliferation Ki-67 (MKI67) and vimentin (VIM) activity while a decreased ECAD signal compared to the DMSO group (Fig. 2H). Additionally, we assessed the effects of D-2HG on TNBC distant metastasis via mice tail vein injection of MDA-MB-231-Luc cells. In vivo bioluminescence and lung tissue imaging revealed that D-2HG enhanced the capacity of MDA-MB-231-Luc cells to metastasize in the lungs (Fig. 2I-M). These results demonstrated that D-2HG promoted TNBC growth and metastasis in vivo.

To elucidate the underlying mechanism by which D-2HG promotes TNBC progression, we performed RNA sequencing (RNA-seq) for transcriptome analysis in D-2HG- or DMSO-treated MDA-MB-468 cells. The heatmap showed 425 upregulated genes and 356 downregulated genes in D-2HG-treated cells (Fig. 2N, Table S2). Both GSVA and KEGG analyses also demonstrated the enrichment of several proliferation and metastasis-related pathways (Fig. 2O-P). The epithelial cell proliferation and epithelial-mesenchymal transition (EMT) pathways were enriched in cells treated with D-2HG (Fig. 2Q), further explaining the morphological changes observed in MDA-MB-468 cells. Western blot assay confirmed that D-2HG upregulated the protein levels related with cell proliferation, migration and invasion in D-2HG-treated TNBC cells (Figure S2H-I). Taken together, our data implied that D-2HG could promote the proliferation, migration, and invasion of TNBC.

### ***ANGPTL4*** is identified as the key gene regulated by D-2HG-mediated m^6^A modification

**Fig. 3:**
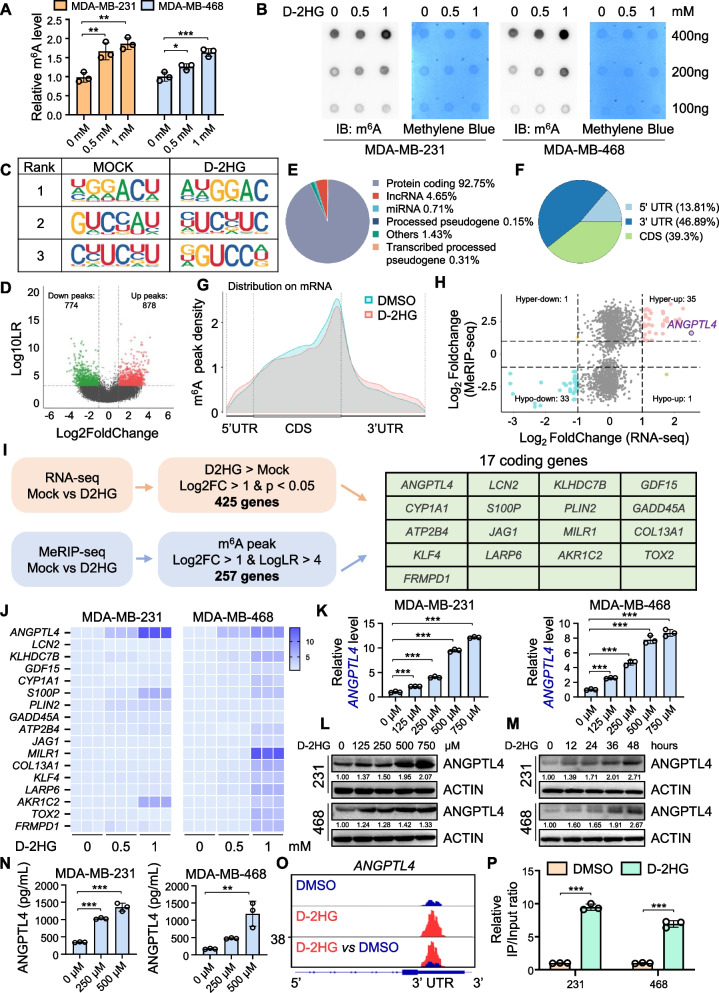
D-2HG increases the total m^6^A level of TNBC cells and promotes *ANGPTL4* expression via m^6^A modification. **A** Treatment with D-2HG elevated global m^6^A levels in TNBC cells. **B** Dot blot analysis was performed to detect the m^6^A methylation levels of poly(A) + mRNA from TNBC cells treated with D-2HG or vehicle, with methylene blue staining serving as a loading control. **C** **S**pecific m^6^A motif analysis of the top three m^6^A motifs in D-2HG- or DMSO-treated MDA-MB-468 cells. **D** Volcano plot of differentially methylated m^6^A peaks between D-2HG and DMSO groups. **E–F** Pie charts display the distribution of differentially methylated m^6^A peaks. **G** Distribution of m^6^A peaks across transcript segments: 5′-untranslated region (UTR), coding sequence (CDS), and 3′-UTR. **H** Dot plot of transcripts exhibiting significant changes in both expression and m^6^A levels following D-2HG treatment. **I** Filtering strategy based on the intersection of upregulated mRNAs and upregulated m^6^A modifications in D-2HG-treated MDA-MB-468 cells (left). The gene list shows the candidate coding genes identified in the intersection (right green table). **J** Heatmaps validating mRNA expression of the candidate coding genes in D-2HG-treated TNBC cells by qPCR. **K** qPCR analysis of *ANGPTL4* in TNBC cells treated with indicated concentrations of D-2HG for 48 h. **L** Same as in K, but with western blot analysis. **M** Western blot analysis of ANGPTL4 in TNBC cells treated with 500 μM D-2HG with indicated different time.** N** Same as in K, but with ELISA analysis. **O** Alterations of m^6^A peaks on *ANGPTL4* transcript in D-2HG- or DMSO-treated MDA-MB-468 cells. **P** Gene-specific m^6^A qPCR validation of *ANGPTL4* m^6^A level changes in TNBC cells. *P < 0.05, **P < 0.01, ***P < 0.001

D-2HG participates in epigenetic modifications by inhibiting various α-KG-dependent dioxygenases, leading to cancer progression through enzyme dysfunction-mediated epigenetic alterations. We observed an overall increase in m^6^A levels in D-2HG-treated cells by m^6^A quantification assay and m^6^A dot blot (Fig. 3A-B). To identify key downstream targets regulated by D-2HG through m^6^A modification, we performed MeRIP-seq on MDA-MB-468 cells treated with D-2HG and DMSO. Both groups exhibited a significant enrichment of the m^6^A consensus motif (GGAC) in the m^6^A peaks, confirming the successful enrichments of m^6^A-modified transcripts (Fig. 3C). We identified 31,905 m^6^A peaks located in 11,892 genes (Table S3), with more peaks (878; 53.1%) displaying increased m^6^A abundance among all the significantly differential peaks (Fig. 3D). The m^6^A peaks were predominantly enriched in protein-coding transcripts (92.75%) (Fig. 3E) and peaked near the stop codon (Fig. 3F-G), consistent with other MeRIP-seq findings. DMSO-treated cells showed reduced overall m^6^A mRNA methylation compared to the D-2HG group (Figure S3A). Mapping these differential m^6^A peaks to human chromosomes revealed significant enrichment on chromosome 1 and chromosome 19 (Figure S3B). Intersection analysis of the genes from RNA-seq and MeRIP-seq, we categorized differentially expressed genes with differential m^6^A methylation into four groups: 35 hyper-up genes, 1 hyper-down gene, 33 hypo-down genes, and 1 hypo-up gene (Fig. 3H). Following the selection criteria, we filtered 17 encoding genes from the 35 hyper-up genes as preliminary candidates for further analysis (Fig. 3I).

We first applied qPCR to determine the expression of the 17 candidate genes in MDA-MB-231 and MDA-MB-468 cell lines treated with D-2HG and found that *ANGPTL4* was among the most significant elevated genes in both two TNBC cell lines (Fig. 3J). We further validated that *ANGPTL4* mRNA expression increased along with D-2HG treatment in a dose- and time-dependent manner (Fig. 3K, Figure S3C). Western blot and immunofluorescence also confirmed that D-2HG promoted ANGPTL4 protein expression (Fig. 3L-M, Figure S3D). As a secreted protein, ANGPTL4 levels in the cell supernatants were detected using ELISA after D-2HG treatment. The results displayed a consistent trend with the western blot findings (Fig. 3N, Figure S3E). Furthermore, *ANGPTL4* was found to be highly expressed in TNBC cell lines compared to non-TNBC cell lines (Figure S3F). The integrative genomics viewer (IGV) visualization of m^6^A peaks at *ANGPTL4* gene locus from MeRIP-seq showed a significant enhancement in the 3’ UTR region after D-2HG treatment, consistent with m^6^A site predictions from the SRAMP database (Fig. 3O, Figure S3G). Gene-specific MeRIP-qPCR confirmed a significant increase in *ANGPTL4* m^6^A levels after D-2HG treatment (Fig. 3P). Previous studies reported that D-2HG could inhibit FTO, an α-KG-dependent dioxygenase, thereby increasing m^6^A RNA modification levels in leukemia cells [35]. We speculated that the increased m^6^A level of *ANGPTL4* in TNBC cells may also rely on the inhibitory effect of D-2HG on FTO. Further data mining of the starBase database for breast cancer (BRCA) samples demonstrated *FTO* expression negatively associated with *ANGPTL4* expression (Figure S3H). We then established *FTO* knockout TNBC cell lines and validated that *FTO* knockout could lead to an increased ANGPTL4 expression, which mimicked the promoting effect of D-2HG on ANGPLT4 expression (Figure S3I-J). MeRIP-qPCR confirmed that *FTO* knockout resulted in a significant increase in m^6^A level of *ANGPTL4* mRNA (Figure S3K). The qPCR and western blot results indicated that FTO overexpression significantly attenuated the effects of D-2HG on ANGPTL4 expression (Figure S3L-M). We then explored whether the increased m^6^A modification of *ANGPTL4* could exert an effect on its mRNA stability. By assessing the *ANGPTL4* RNA decay rates in *FTO* knockout TNBC cells and control cells (Figure S3N), we found that *FTO* knockout increased ANGPTL4 mRNA stability in both TNBC cell lines. Based on these results, we found that ANGPTL4 is a crucial gene regulated by D-2HG-mediated m^6^A modification in TNBC.

### YTHDF1 facilitates ANGPTL4 translation in an m^6^A-dependent manner

**Fig. 4:**
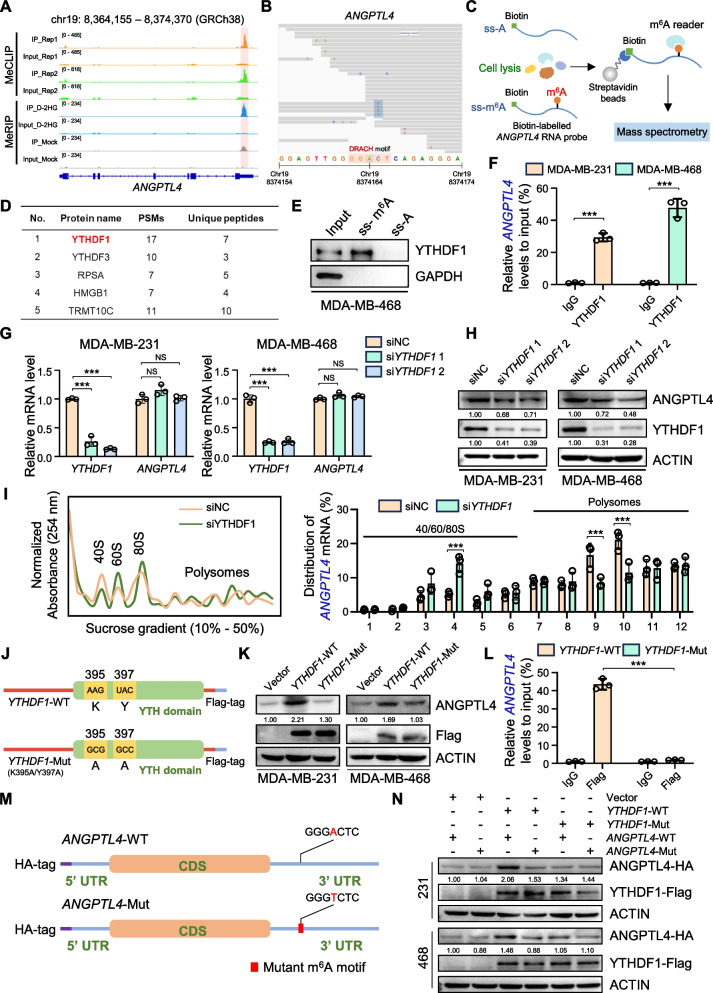
YTHDF1 enhances ANGPTL4 translation via an m^6^A-dependent manner. **A** Distribution of m^6^A-binding peaks (MeCLIP-seq from GSE147440 and MeRIP-seq) across the *ANGPTL4* transcript. **B** Genome browser snapshot of the m^6^A residue in *ANGPTL4* (GSE147440). The red box surrounding the “A” indicates the m^6^A site (chr19: 8,374,164), with the C-to-T mutations at the downstream “C” depicted in the reads (blue bars) aligning to that locus. **C** Schematic diagram of the procedure for screening *ANGPTL4* mRNA m^6^A readers using RNA pull-down and mass spectrometry with *ANGPTL4* m^6^A site-specific methylated single‐stranded RNA (ss‐m^6^A) or unmethylated control RNA (ss‐A) probes. **D** Candidate proteins binding to the *ANGPTL4* ss-m^6^A probes identified by mass spectrometry. **E** Western blot analysis of YTHDF1 in MDA-MB-468 cells following the RNA pull-down assay. **F** YTHDF1-specific RIP-derived RNA from MDA-MB-231 and MDA-MB-468 cells was measured by qPCR. **G** Relative RNA levels of *ANGPTL4* in MDA-MB-231 and MDA-MB-468 cells upon *YTHDF1* knockdown. **H** Western blot analysis detected the protein levels of ANGPTL4 in MDA-MB-231 and MDA-MB-468 cells upon *YTHDF1* knockdown. **I** Polysome profiling of MDA-MB-468 cells following *YTHDF1* knockdown (left), and relative *ANGPTL4* mRNA distribution in each ribosome fraction analyzed by qPCR (right). **J** Schematic description of wild-type (*YTHDF1*-WT) and mutant (*YTHDF1*-Mut) constructs of YTHDF1. **K** Western blot analysis of ANGPTL4 in TNBC cells transfected with *YTHDF1*-WT or *YTHDF1*-Mut. **L** Flag-specific RIP-derived RNA in MDA-MB-468 cells was measured by qPCR. **M** Schematic description of wild-type (*ANGPTL4*-WT) and mutant (*ANGPTL4*-Mut) constructs of ANGPTL4. **N** Western blot analysis of ANGPTL4 expression in MDA-MB-231 and MDA-MB-468 cells transfected with the empty vector, Flag-tagged *YTHDF1*-WT/-Mut, and HA-tagged *ANGPTL4*-WT/-Mut. *P < 0.05, **P < 0.01, ***P < 0.001, NS, no significance

To further elucidate the underlying molecular mechanism by which m^6^A modification regulates ANGPTL4 expression, we first performed a combined analysis of results from MeRIP-seq and MeCLIP-seq (GSE147440), confirming that the m^6^A peaks were enriched in the 3’UTR region of *ANGPTL4* (Fig. 4A). The “A” base at position chr19: 8,374,164 was identified as a high-confidence m^6^A site due to its distinct "cliff" pattern observed in the MeCLIP-seq data (Fig. 4B). The site was also predicted as the only "very high confidence" site in the SRAMP database (Figure S3G, Table S4). Since m^6^A modification regulates post-transcriptional gene expression primarily through specific m^6^A readers [36], we aimed to identify the key m^6^A reader that regulates ANGPTL4 expression via D-2HG-mediated m^6^A modification in TNBC.

We used biotin-labeled ssRNA probes, with or without m^6^A methylation modification (ss-A or ss-m^6^A), to perform RNA pull-down and mass spectrometry with MDA-MB-468 cell lysates (Fig. 4C, Figure S4A). We observed significant enrichment of YTHDF1 among proteins specifically bound to ss-m^6^A probes (Fig. 4D, Figure S4B, Table S5). RNA pull-down and western blot experiments confirmed YTHDF1 selectively bound to *ANGPTL4* ss-m^6^A probes in MDA-MB-468 cells (Fig. 4E). The specific interaction between YTHDF1 protein and *ANGPTL4* mRNA was further verified by RIP assay in TNBC cell lines (Fig. 4F, Figure S4C-D). As one of the key m^6^A readers, YTHDF1 has been reported to influence ribosome occupancy and translation of m^6^A-modified mRNAs [11]. To explore how YTHDF1 regulates ANGPTL4 expression in TNBC cells, we knocked down *YTHDF1* with small interfering RNAs (siRNAs), resulting in a significant decrease in ANGPTL4 protein levels without notable changes in RNA levels (Fig. 4G-H). Linkedomics database investigation showed no significant RNA-level correlation between *ANGPTL4* and *YTHDF1* (Figure S4E), suggesting that YTHDF1 might regulate the translation efficiency or stability of the ANGPTL4 protein. We then assessed the regulation of ANGPTL4 expression levels following *YTHDF1* knockdown under the protein synthesis inhibitor CHX treatment. The results indicated that *YTHDF1* knockdown did not significantly affect ANGPTL4 protein stability in TNBC cells under CHX conditions (Figure S4F). Conversely, the ribosome profiling assay revealed that *YTHDF1* knockdown resulted in a reduction in *ANGPTL4* mRNA in actively translating polysomes, resulting in a shift to non-polysome fractions (Fig. 4I, Figure S4G). This suggests that YTHDF1 regulates ANGPTL4 protein synthesis rather than protein stability.

The YTH domain is essential for YTHDF1 recognition and binding to the m^6^A site, and mutations at K395 and Y397 on YTH domain hinder its binding ability [37]. We introduced the point mutations K395A and Y397A (*YTHDF1*-Mut) into the YTH domain of the YTHDF1 expression vector with a Flag tag (Fig. 4J). Transfection of TNBC cell lines with either *YTHDF1*-WT or *YTHDF1*-Mut showed that *YTHDF1*-WT, but not *YTHDF1*-Mut, increased ANGPTL4 protein expression (Fig. 4K). Afterwards, RIP using the anti-Flag antibody followed by qPCR showed that *ANGPTL4* mRNA was effectively enriched in *YTHDF1*-WT immunoprecipitates, while was significantly reduced in the *YTHDF1*-Mut samples (Fig. 4L, Figure S4H). These results indicated that the YTH domain of YTHDF1 is crucial for recognizing and binding to the m^6^A site in *ANGPTL4* mRNA. Additionally, we constructed an HA-tagged full-length *ANGPTL4* mRNA expression vector (*ANGPTL4*-WT) and m^6^A site-mutated vector (*ANGPTL4*-Mut) (Fig. 4M) to further confirm the binding site. Western blot analysis showed *YTHDF1*-WT, but not *YTHDF1*-Mut, enhanced *ANGPTL4*-WT expression, with minimal impact on *ANGPTL4*-Mut expression (Fig. 4N). To validate whether the effect of D-2HG on ANGPTL4 expression is dependent on YTHDF1, we treated *YTHDF1*-silenced TNBC cells with D-2HG. The ability of D-2HG to enhance ANGPTL4 expression was diminished in *YTHDF1*-silenced cells (Figure S4I). These results collectively demonstrate that YTHDF1 promotes ANGPTL4 translation in an m^6^A-dependent manner.

### Knockdown of *ANGPTL4* attenuates D-2HG-induced proliferation and metastasis of TNBC cells via the integrin pathway

**Fig. 5:**
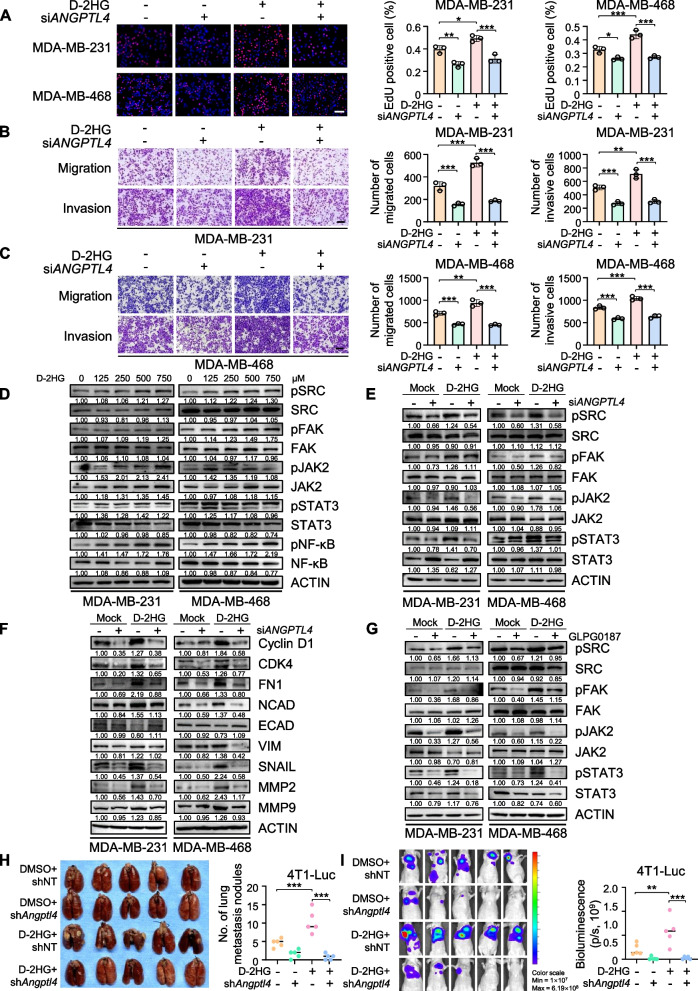
*ANGPTL4* knockdown attenuates the oncogenic effect of D-2HG in TNBC cells via integrin-regulated pathway. **A** Representative images and quantification of the EdU assay in TNBC cells. Scale bar: 100 μm. **B** Representative images and quantification of migration and invasion assays in MDA-MB-231 cells. Scale bar: 200 μm. **C** Representative images and quantification of migration and invasion assays in MDA-MB-468 cells. Scale bar: 200 μm. **D** TNBC cells were treated with D-2HG for the indicated concentration, and activation of the integrin/SRC/FAK/JAK2/STAT3 pathway was evaluated by western blot. **E** Western blot analysis of integrin/SRC/FAK/JAK2/STAT3 pathway expression in *ANPGTL4* knockdown TNBC cells following D-2HG treatment (500 μM) for 48 h. **F** Western blot analysis of expression of proteins involved in cell cycle, migration, and invasion in *ANPGTL4* knockdown TNBC cells following D-2HG treatment (500 μM) for 48 h. **G** TNBC cells were pre-treated with 5 ng/mL GLPG0187 for 12 h, followed by D-2HG treatment (500 μM) for 48 h, western blot showed the protein expression related to the integrin pathway. **H** Images (left) and quantification (right) of lung metastatic nodules from BALB/c nude mice at the endpoint (*n* = 5/group). **I** BLI images and quantification (right) of BALB/c nude mice intravenously injected with *Angptl4* knockdown 4T1 cells followed by administration of D-2HG or vehicle (*n* = 5/group). *P < 0.05, **P < 0.01, ***P < 0.001

To explore whether ANGPTL4 plays a key role in D-2HG-induced proliferation and metastasis in the TNBC cells, we characterized the cellular phenotypes with D-2HG treatment in *ANGPTL4*-silenced cells. Both qPCR and western blot showed that *ANGPTL4* was efficiently knocked down by siRNAs (Figure S5A-B). EdU and transwell assays demonstrated *ANGPTL4* knockdown significantly attenuated the promotive effects of D-2HG on TNBC cell proliferation and metastasis (Fig. 5A-C). Previous studies have shown that secreted ANGPTL4 interacts with integrin β1 and β5 on the cell surface, triggering downstream signaling pathways such as focal adhesion kinase (FAK) and proto-oncogene tyrosine-protein kinase SRC, thereby affecting tumor progression [38, 39]. Our results further validated that as the concentration and the exposure time of D-2HG increased, there was a more pronounced activation of the integrin signaling pathway (Fig. 5D, Figure S5C-D). Furthermore, KEGG and GSEA analysis of the RNA-seq data shown in Fig. 2 revealed the activation of the JAK-STAT3 pathway (Fig. 2O, Figure S5E), which has been reported to be the downstream pathway of activated FAK and SRC [40–42]. We also observed the activation of JAK2-STAT3 and NF-kB pathways under D-2HG treatment. Furthermore, we validated that the knockdown of *ANGPTL4* could markedly inhibit the D-2HG-induced activation of the integrin and downstream pathways (Fig. 5E) and also confirmed that *ANGPTL4* knockdown mitigated the effect of D-2HG on cell cycle progression and EMT process in TNBC cells (Fig. 5F), suggesting that D-2HG-induced ANGPTL4 could bind to integrin proteins in TNBC tumor cells and activate a series of downstream oncogenic signaling pathways, thus promoting TNBC progression.

Integrin proteins are potential therapeutic targets in solid tumors, with ongoing studies involving antibodies, synthetic mimic peptides, antibody–drug conjugates, etc. [43]. Cells were treated with the integrin antagonist GLPG0187, followed by exposure to D-2HG as indicated. EdU, transwell, and western blot assays demonstrated that inhibiting the integrin pathway effectively attenuated the oncogenic effects of D-2HG in TNBC cells (Fig. 5G, Figure S5F-I). Taken together, our results demonstrated that D-2HG-induced elevation of ANGPTL4 activates integrin/FAK/SRC/JAK2/STAT3 pathway, thereby promoting oncogenic effects in TNBC.

### D-2HG-mediated ANGPTL4 secretion promotes macrophage M2 polarization in the tumor microenvironment

**Fig. 6:**
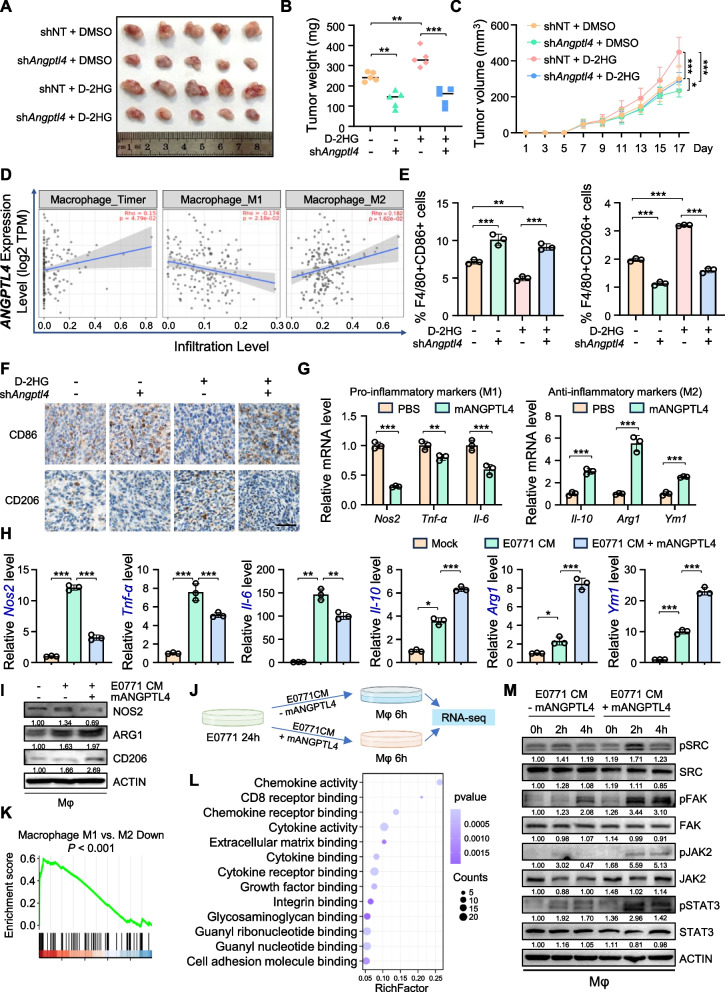
D-2HG induces ANGPTL4 secretion to enhance macrophage M2 polarization in the tumor microenvironment. **A-C** 4T1 shNT cells or sh*Angptl4* cells were subcutaneously injected into nude mice, with D-2HG administered intraperitoneally on days 7, 9, 11, 13, and 15. Tumors were dissected on day 17 and photographed (A). Tumor weight (B) and growth curve (C) were analyzed (*n* = 5/group). **D** Correlation between macrophage infiltration and *ANGPTL4* expression (TIMER database). **E** Flow cytometry analysis of macrophage subpopulations in 4T1 xenografts. The percentages of M1-like (F4/80 + CD86 +) and M2-like (F4/80 + CD206 +) tumor-infiltrating macrophages were calculated (*n* = 3/group). **F.** Representative images of CD86 and CD206 immunohistochemical staining in tumor sections. Scale bar: 50 μm. **G** Inflammation-related marker genes of peritoneal macrophages were analyzed by qPCR after stimulation with recombinant mouse ANGPTL4 protein (mANGPTL4) or PBS for 24 h. **H** Inflammation-related marker genes of peritoneal macrophages were analyzed by qPCR after treatment with E0771 conditioned media (CM) and 100 ng/mL mANGPTL4 or PBS for 6 h. **I** Inflammation-related marker genes of peritoneal macrophages were analyzed by western blot after E0771 CM treatment with 100 ng/mL mANGPTL4 or PBS for 24 h. **J** Schematic diagram of RNA-seq for exploring the role of ANGPTL4 in regulating macrophage polarization. **K** GSEA analysis of RNA-seq results showed the enhanced enrichment of the macrophage M1 vs. M2 down pathway in E0771 CM-treated peritoneal macrophages combined with mANGPTL4. **L** KEGG analysis showed ANGPTL4 was involved in the regulations of pathways related to cytokine and chemokine binding. **M** Activation of the integrin pathway in peritoneal macrophages was analyzed by western blot after E0771 CM treatment with 100 ng/mL mANGPTL4 or PBS for the indicated duration. *P < 0.05, **P < 0.01, ***P < 0.001

To further evaluate whether the knockdown of *ANGPTL4* in TNBC cells can inhibit the oncogenic effects of D-2HG in vivo, we applied tumorigenesis and lung metastasis models. We first validated that *Angptl4* expression upregulated with the increased D-2HG concentration and exposure time in mouse TNBC cell lines 4T1 and E0771 (Figure S6A-D). We then established stable *Angptl4*-knockdown 4T1 cell lines, confirmed at both mRNA and protein levels (Figure S6E), and injected these knockdown and control cells subcutaneously into BALB/c nude mice. After 7 days of tumor formation, the mice were divided into D-2HG and DMSO treatment groups. Ten days after treatment, the mice were sacrificed, and tumors were isolated. Tumors in the *Angptl4* knockdown group grew slower than those in the control group and did not show significant enlargement even after D-2HG treatment (Fig. 6A-C). Next, we injected 4T1-Luc cells with *Angptl4* knockdown and control cells into the tail veins of BALB/c nude mice. After four weeks, the mice were administered with D-2HG or DMSO and observed for lung metastasis after six weeks. Injection of *Angptl4* knockdown cells reduced the lung metastasis ability of 4T1 cells and did not increase lung metastasis even after D-2HG treatment (Fig. 5H-I). In summary, *Angptl4* knockdown impaired tumorigenesis and metastatic effects of D-2HG in TNBC.

In addition to its effects on tumor cells, we investigated whether ANGPTL4 could impact the TME in TNBC. ANGPTL4 plays a crucial role in immune regulation, particularly in inflammation modulation. Previous studies on peritonitis and myocardial infarction have demonstrated that ANGPTL4 primarily exhibits anti-inflammatory effects by reducing inflammatory cell infiltration and pro-inflammatory cytokine release [44, 45]. Using the TIMER database, we analyzed the relationship between *ANGPTL4* expression and immune infiltration, noting that *ANGPTL4* expression was inversely correlated with M1-like macrophage levels and positively correlated with M2-like macrophage infiltration in basal-like BRCA (Fig. 6D). Therefore, we assessed macrophage polarization in solid tumors (as shown in Fig. 6A) using flow cytometry and immunohistochemistry. Compared to the control group, the knockdown of *ANGPTL4* promoted M1 polarization and inhibited M2 polarization, which could not be rescued by the administration of D-2HG (Fig. 6E-F, Figure S6F-H). These findings indicated that ANGPTL4 induced by D-2HG could regulate the polarization of tumor-infiltrating macrophages. The peritoneal macrophages isolated from C57 mice exhibited decreased expression of pro-inflammatory *Nos2*, *Tnf-α*, and* Il-6* (M1 markers) and increased expression of anti-inflammatory* Il-10*, *Arg1*, and *Ym1* (M2 markers) after treatment with recombinant mANGPTL4 (Fig. 6G). Using E0771 conditioned medium (E0771 CM) combined with or without recombinant mANGPTL4 stimulation, we further confirmed that ANGPTL4 promoted M2 polarization and inhibited M1 polarization (Fig. 6H-I).

To elucidate the molecular mechanism of ANGPTL4 on macrophage polarization, we performed RNA-seq on E0771 CM-treated cells with or without recombinant mANGPTL4 (Fig. 6J). GSEA analysis indicated that ANGPTL4 could promote M2-like polarization in macrophages and significantly inhibit various immune-related pathways (Fig. 6K, Figure S6I), consistent with previous findings supporting the anti-inflammatory activity of ANGPTL4. KEGG enrichment analysis further demonstrated that ANGPTL4 regulated pathways related to cytokine and chemokine binding, particularly the integrin binding pathway (Fig. 6L). We speculated that ANGPTL4 might promote M2 polarization of macrophages through activation of the integrin pathway. Western blot results confirmed that ANGPTL4 enhanced the activation of the integrin and its downstream pathways in E0771 CM-treated cells, leading to M2 polarization of macrophages (Fig. 6M). In summary, D-2HG-mediated secretion of ANGPTL4 promotes M2 polarization of tumor-associated macrophages in the TME, inhibiting the anti-tumor immune responses and promoting TNBC growth and metastasis.

### ANGPTL4 can serve as a specific prognostic biomarker for TNBC

**Fig. 7:**
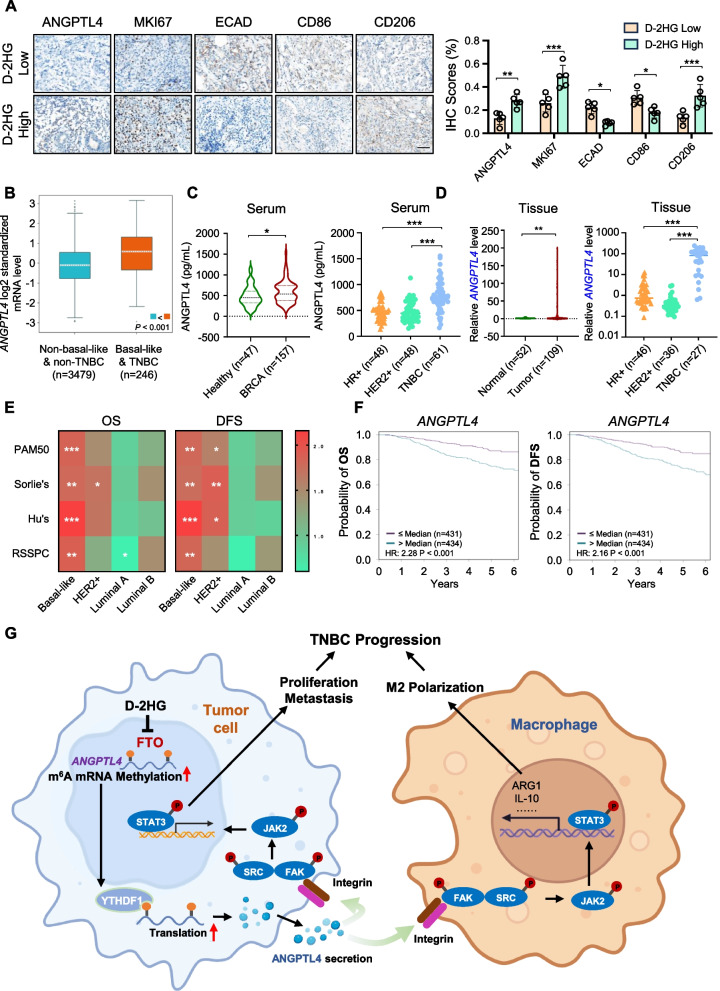
ANGPTL4 is associated with poor prognosis in TNBC patients.** A** Representative images (left) and quantification (right) of ANGPTL4, MKI67, ECAD, CD86 and CD206 expression in TNBC specimens with high or low D-2HG levels. Scale bar: 50 μm. **B** Box and whisker plot of *ANGPTL4* expression according to basal-like and TNBC status from the Breast Cancer Gene-Expression Miner (bc-GenExMiner) database. **C** Violin plot (left) and scatter plot (right) of ANGPTL4 levels in the serum of healthy controls or BRCA patients from the Qilu cohort. **D** Violin plot (left) and scatter plot (right) of *ANGPTL4* mRNA levels in BRCA tumor tissues versus normal tissues from the Qilu cohort. **E** Heatmaps showing the correlations between *ANGPTL4* expression and overall survival (OS) as well as disease-free survival (DFS), based on the hazard ratio (HR) from different molecular subtype classification methods in bc-GenExMiner. **F** Survival curves of basal-like patients according to Hu’s breast cancer classification method in bc-GenExMiner for the correlations between *ANGPTL4* expression and OS as well as DFS. **G** Schematic diagram underlying the roles of D-2HG-mediated m^6^A-dependent ANGPTL4 secretion in TNBC, which facilitates tumor progression by regulating tumor cell proliferation and metastasis (via autocrine signaling), as well as enhancing tumor-promoting M2 macrophage infiltration (via paracrine signaling). *P < 0.05, **P < 0.01, ***P < 0.001

Based on the mechanisms described above, we further explored the clinical significance of ANGPTL4 in breast cancer and its correlation with D-2HG. We first performed IHC detection on TNBC tissues with high and low accumulation of D-2HG. The results indicated that patients with high levels of D-2HG exhibited higher expression of ANGPTL4, MKI67, and CD206, and lower expression of ECAD and CD86 (Fig. 7A). Using the breast cancer online database bc-GenExMiner, box plots indicated that *ANGPTL4* expression at the RNA level was significantly elevated in basal-like and TNBC subtypes (Fig. 7B). ELISA measurements of serum samples showed that ANGPTL4 was most prominently secreted in the serum of BRCA patients, especially in that of TNBC patients (Fig. 7C). The qPCR analysis of patients’ tissues yielded similar results (Fig. 7D). Furthermore, database analysis revealed that regardless of the subtype classification strategies used, increased *ANGPTL4* expression was substantially linked to poor overall survival (OS) and disease-free survival (DFS) in basal-like subtypes (Fig. 7E). Survival curves further indicated that high *ANGPTL4* expression in TNBC patients was significantly associated with a poor prognosis (HR = 2.28, *P* < 0.001, HR = 2.16, *P* < 0.001, respectively, Fig. 7F). Taken together, the elevated levels of ANGPTL4 significantly existed in both the serum and tissues of TNBC patients, and this increased expression was correlated with poor outcomes, suggesting that ANGPTL4 could serve as a high-performance prognostic biomarker for TNBC patients.

## Discussion

In this study, we provide compelling evidence for the accumulation of D-2HG in breast cancer, with particular emphasis on TNBC. Our research demonstrates that D-2HG-induced m^6^A modification of ANGPTL4 exerts a dual effect: it not only promotes tumor cell proliferation and metastasis but also modulates macrophage polarization within TME. These effects are mediated through the ANGPTL4-activated integrin/FAK/SRC/JAK2/STAT3 signaling cascade (Fig. 7G). Importantly, our findings are substantiated by evidence from patient-derived organoids, mouse models, and clinical samples, underscoring the clinical relevance of the D-2HG-ANGPTL4 axis. This study elucidates the mechanistic role of D-2HG in the m^6^A modification of ANGPTL4, thereby identifying a potential therapeutic target for TNBC.

Metabolic reprogramming equips cancer cells with the capacity to grow, proliferate, and survive within the nutrient-deprived TME. These metabolic alterations lead to the accumulation of specific oncogenic metabolites, commonly termed oncometabolites. Among these, 2HG is one of the earliest identified oncometabolites, with its accumulation documented across various cancer types [46]. Our research, along with that of others, has demonstrated that D-2HG accumulates in breast cancer, particularly in TNBC, in both clinical tissue samples and cell lines [17, 47, 48]. Notably, breast cancer seldom exhibits mutations in *IDH1* and *IDH2*, which are known to drive 2HG accumulation in gliomas and leukemia [46], suggesting alternative mechanisms are responsible for the elevated 2HG levels observed in breast cancer. In breast cancer, the amplification of *PHGDH* and upregulation of ADHFE1 have been implicated in the accumulation of D-2HG [33, 49]. Our study reveals a significant elevation of D-2HG levels in the serum and tumor tissues of TNBC, potentially regulated by high PHGDH expression and low D2HGDH expression, both of which correlate with poor prognosis in breast cancer.

D-2HG has long been recognized as a modulator of epigenetic modifications, primarily due to its critical role in inhibiting α-KG-dependent dioxygenases involved in histone and DNA demethylation, such as TET enzymes [50], KDM histone demethylases [51], and AlkB family [52], leading to widespread epigenetic alterations. However, its influence on RNA epigenetic modifications has been relatively underexplored. Emerging evidence suggests that m^6^A modification, the most prevalent RNA epigenetic modification in eukaryotes, is essential for the progression of several types of cancer, including renal cell carcinoma, hepatocellular carcinoma, and colorectal cancer [53–55], by modulating the expression of oncogenes and tumor suppressor genes. Nevertheless, the specific role of m^6^A modification in breast cancer, particularly in TNBC, remains a relatively new and largely uncharted area. In our study, we identified that D-2HG accumulation inhibited the activity of FTO, leading to increased m^6^A modification of *ANGPTL4*, a protein known to promote tumor progression and metastasis [44, 56, 57]. It is well-established that alterations in m^6^A levels of target genes, and their subsequent expression, require the intermediary participation of m^6^A "reader" proteins. In the context of breast cancer, reported m^6^A readers include the YTH, HNRNP, FXR, and the IGF2BP family [58]. Our research reveals that the m^6^A "reader" YTHDF1 enhances ANGPTL4 translation by binding to the m^6^A-modified 3' UTR of *ANGPTL4* mRNA, thereby promoting its translation. Notably, mutation at the m^6^A modification sites of *ANGPTL4* reduces its binding affinity to YTHDF1. The mechanistic connection between D-2HG and ANGPTL4 expression via m^6^A modification offers novel insights into the epigenetic regulation of cancer metabolism.

Our research demonstrates that D-2HG not only accumulates in TNBC but also plays an active role in promoting tumor progression. In vitro and in vivo experiments indicated that D-2HG enhanced the proliferation, migration, and invasion of TNBC cells, primarily through the ANGPTL4-induced activation of the integrin signaling pathway. These findings underscore the role of D-2HG as a potent oncogenic metabolite in TNBC. Besides this, we further investigated its function in TME since ANGPTL4 was reported as a secretory protein. Currently, there are limited studies reporting how ANGPTL4 regulates TME in breast cancer. In the brain metastatic microenvironment, TGF-β2 secreted by astrocytes stimulates the upregulation of ANGPTL4 expression in TNBC cells, inducing the SMAD signaling pathway to promote TNBC brain metastasis [59]. Activation of the STAT3 pathway in cancer-associated fibroblasts leads to elevated secretion of ANGPTL4, which is associated with shorter survival in patients [57]. Macrophage polarization plays a crucial role in shaping the TME. M1 macrophages exhibit pro-inflammatory and anti-tumor properties, whereas M2 macrophages support tumor growth and immune evasion [60]. Our study is the first to report that ANGPTL4 encourages the polarization of tumor-infiltrating macrophages toward the M2 phenotype within the TME, highlighting the multifaceted role of ANGPTL4 in promoting tumor progression.

Due to its rapid progression, high metastatic capability, and resistance to conventional treatments, TNBC is widely recognized as the most aggressive and challenging subtype of breast cancer [61], underscoring the urgent need to identify effective therapeutic targets to overcome this challenging disease. Given the central role of D-2HG in the progression of TNBC, targeting the pathways associated with its accumulation presents promising therapeutic opportunities. Current clinical trials targeting elevated D-2HG levels, particularly in cancers with *IDH* mutations such as gliomas, acute myeloid leukemia, and chondrosarcomas, have demonstrated favorable responses to *IDH-*mutant selective inhibitors [62–64]. However, cancers with elevated D-2HG levels in the absence of *IDH* mutations continue to pose significant therapeutic challenges. Our findings suggest that patients with high levels of D-2HG tend to exhibit elevated expression of ANGPTL4, which is markedly upregulated in TNBC. This highlights ANGPTL4 as a potential specific prognostic marker and a novel therapeutic target for TNBC patients with elevated D-2HG levels. Future translational research can focus on developing ANGPTL4 inhibitors, including designing neutralizing antibodies to block its function, antisense oligonucleotides to induce gene silencing, and small-molecule inhibitors to suppress its activity or expression. Combining ANGPTL4 inhibitors with other therapies, such as chemotherapy or immune checkpoint inhibitors, is also a promising approach worth exploring for TNBC.

Despite the significant findings presented in our study, several limitations must be acknowledged. Considering the heterogeneity and variability of the TNBC TME, the oncogenic role of ANGPTL4 via M2 macrophage polarization may not fully capture the intricate dynamics of immune modulation in TNBC patients. Consequently, translating these findings into clinical settings will necessitate further validation using a larger cohort of clinical samples. Additionally, the accumulation of D-2HG is influenced by a variety of enzymes, and the genes and pathways modulated by D-2HG warrant further in-depth investigation in TNBC and other cancers.

## Conclusions

Collectively, this study underscores the oncogenic role of D-2HG in TNBC, with its effects mediated through epigenetic regulation of the critical gene *ANGPTL4*. These findings offer a strong foundation for the development of targeted therapies aimed at D-2HG and its downstream signaling pathways, potentially enhancing treatment outcomes in patients with aggressive TNBC.

## Supplementary Information

Supplementary Material 1. Supplementary Table 1. Key reagents, resources and sequences of primers used in qPCR, RIP-qPCR, MeRIP-qPCR, siRNA/shRNA target oligo, and probes for RNA pull down assay used in this study.

Supplementary Material 2. Supplementary Table 2. RNA-seq analysis results of MDA-MB-468 cells treated with D-2HG and DMSO.

Supplementary Material 3. Supplementary Table 3. MeRIP-seq analysis results of MDA-MB-468 cells treated with D-2HG and DMSO.

Supplementary Material 4. Supplementary Table 4. List of potential m^6^A sites in the human *ANGPTL4* transcript predicted using SRAMP.

Supplementary Material 5. Supplementary Table 5. Proteins that specifically bind to *ANGPTL4 *ss- m^6^A probes identified by RNA pull-down assay.

Supplementary Material 6.

## Data Availability

The datasets used and analysed during the current study are available from the corresponding author on reasonable request.

## Abbreviations

D-2HG: D-2-hydroxyglutarate
TNBC: Triple-negative breast cancer
m^6^A: N6-methyladenosine
FTO: Fat-mass and obesity-associated protein
ANGPTL4: Angiopoietin Like 4
EMT: Epithelial-mesenchymal transition
TCGA: The Cancer Genome Atlas
YTHDF1: YTH N6-methyladenosine RNA binding protein F1
OS: Overall survival
DFS: Disease-free survival
MeRIP: Methylated RNA immunoprecipitation
MeCLIP: M^6^A enhanced cross-linking and immunoprecipitation
qPCR: Quantitative PCR
ELISA: Enzyme-linked immunosorbent assay
IDH: Isocitrate dehydrogenase
PHGDH: Phosphoglycerate dehydrogenase
D2HGDH: D-2-hydroxyglutarate dehydrogenase
ssRNA: Single-stranded RNA
TME: Tumor microenvironment
FAK: Focal adhesion kinase
MKI67: Marker of proliferation Ki-67
ECAD: E-cadherin
VIM: Vimentin
