# Supplementary material for: Oncometabolite D-2HG drives tumor metastasis and protumoral macrophage polarization by targeting FTO/m6A/ANGPTL4/integrin axis in triple-negative breast cancer
Source: J Exp Clin Cancer Res. 2025 Feb 6;44:41. doi: 10.1186/s13046-025-03282-1 (PMC11800637; doi:10.1186/s13046-025-03282-1)
Supplement: Supplementary file 6 — Supplementary Material 6. [file 13046_2025_3282_MOESM6_ESM.docx]

**Supplementary figures and figure legends**


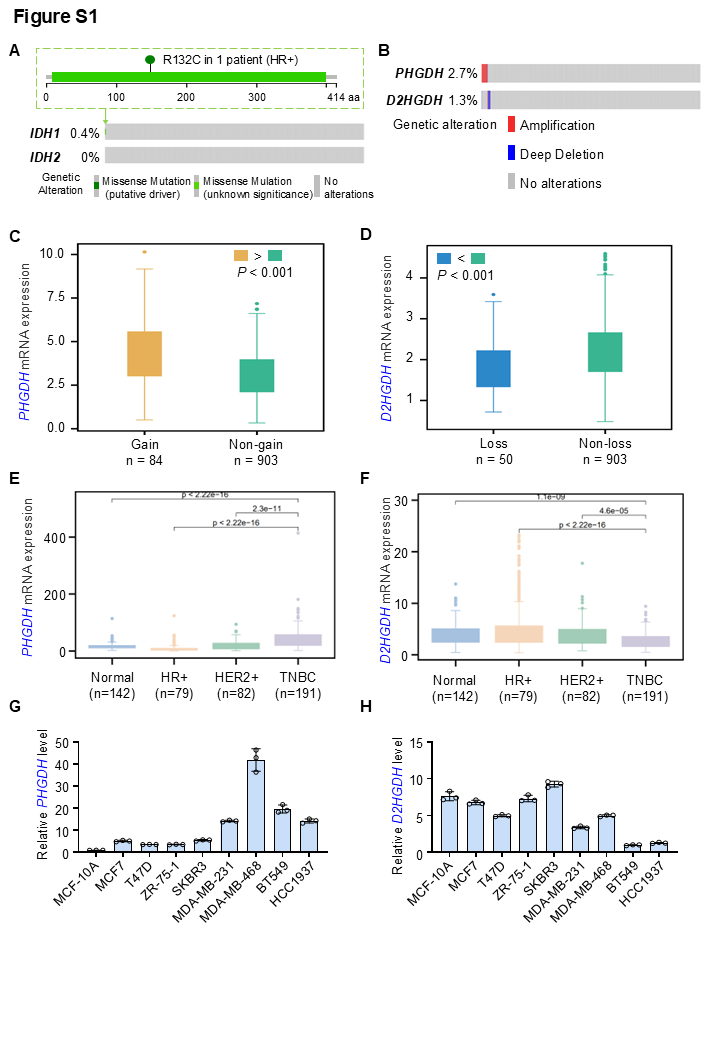


**Figure S1. D-2HG accumulation depends on overexpression of PHGDH and downregulation of D2HGDH in TNBC. A.** Analysis of *IDH1/2* mutations in BRCA patients from TCGA database. **B.** Amplification and depletion analysis of *PHGDH* and *D2HGDH* in BRCA patients from TCGA database. **C.** Correlation between the expression of *PHGDH* with its gene amplification status in BRCA patients from TCGA database. **D.** Correlation between the expressions of *D2HGDH* with its gene deletion status in BRCA patients from TCGA database. **E.** Box plot of *PHGDH* mRNA levels in tumor versus normal samples from TCGA data. **F.** Box plot of *D2HGDH* mRNA levels in tumor versus normal samples from TCGA data. **G.** qPCR analysis of *PHGDH* mRNA expression in breast cancer cell lines. **H.** qPCR analysis of *D2HGDH* mRNA expression in breast cancer cell lines.


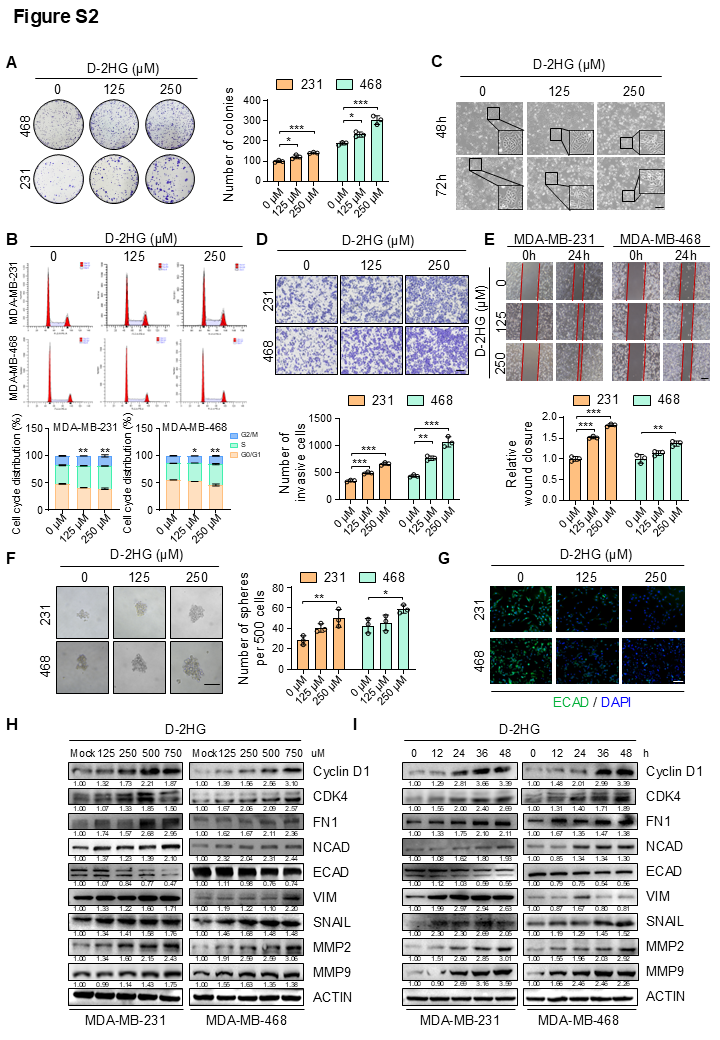


**Figure S2. D-2HG enhances cell growth and invasiveness of TNBC cells. A.** Representative images and quantification of the colony formation assay in TNBC cells. **B.** Cell cycle assay of D-2HG-treated TNBC cells using flow cytometry. **C.** MDA-MB-468 cells treated with D-2HG exhibited a mesenchymal phenotype. Scale bar: 200 μm. **D.** Representative images and quantification of the invasion assay in TNBC cells. Scale bar: 200 μm. **E.** Representative images and quantification of the wound healing assay in TNBC cells. Scale bar: 200 μm. **F.** Representative images and quantification of the sphere formation assay in TNBC cells. Scale bar: 100 μm. **G.** Representative images of immunofluorescence assay detecting ECAD expression in TNBC cells with or without D-2HG treatment. Scale bar: 100 μm. **H.** Western blot analysis showed the effect of D-2HG on the expression of proteins involved in cell cycle, migration, and invasion in TNBC cells treated with D-2HG for varying concentrations, as indicated.**I.** Western blot analysis showed the effect of D-2HG on the expression of proteins involved in cell cycle, migration, and invasion in TNBC cells treated with 500 μM D-2HG for different durations, as indicated. **P* < 0.05, ***P* < 0.01, ****P* < 0.001.


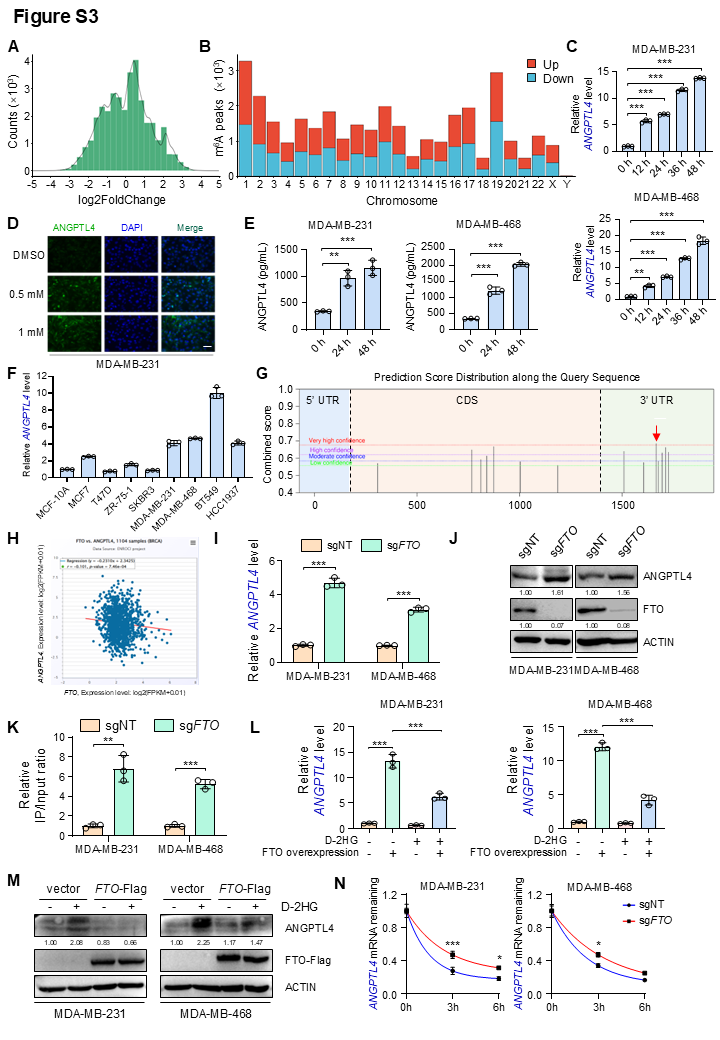
  **Figure S3. Identification of D-2HG-mediated m^6^A-modified transcripts in TNBC cells. A.** Histogram showing the changes in m^6^A enrichment between D-2HG and DMSO treated MDA-MB-468 cells. **B.** Distribution of differentially methylated m^6^A peaks (fold change ≥ 2) in human chromosomes. **C.** qPCR analysis of *ANGPTL4* expression in TNBC cells treated with 500 μM D-2HG with indicated time. **D.** Representative images of immunofluorescence assay for ANGPTL4 expression in MDA-MB-231 cells with or without D-2HG treatment. Scale bar: 100 μm. **E.** ELISA analysis of *ANGPTL4* expression in TNBC cells treated with 500 μM D-2HG with indicated time. **F.** qPCR analysis of *ANGPTL4* mRNA expression in breast cancer cell lines. **G.** Predicted potential m6A modification sites in *ANGPTL4* mRNA using SRAMP website. **H.** Correlation between the expression of *FTO* and *ANGPTL4* (starBase database). **I.** Relative *ANGPTL4* mRNA levels in *FTO* knockout and control MDA-MB-231 and MDA-MB-468 cells. **J.** Relative protein levels of ANGPTL4 in *FTO* knockout versus control MDA-MB-231 and MDA-MB-468 cells. **K.** Gene-specific m^6^A qPCR validation of *ANGPTL4* m^6^A level changes in TNBC cells. **L-M.** qPCR (L) and western blot (M) analysis of protein expression of ANGPTL4 in *FTO* overexpression TNBC cells after D-2HG treatment for 48h. **N.** The mRNA stability of *ANGPTL4* was assessed by qPCR in *FTO* knockout and control TNBC cells following Actinomycin D treatment. **P* < 0.05, ***P* < 0.01, ****P* < 0.001.


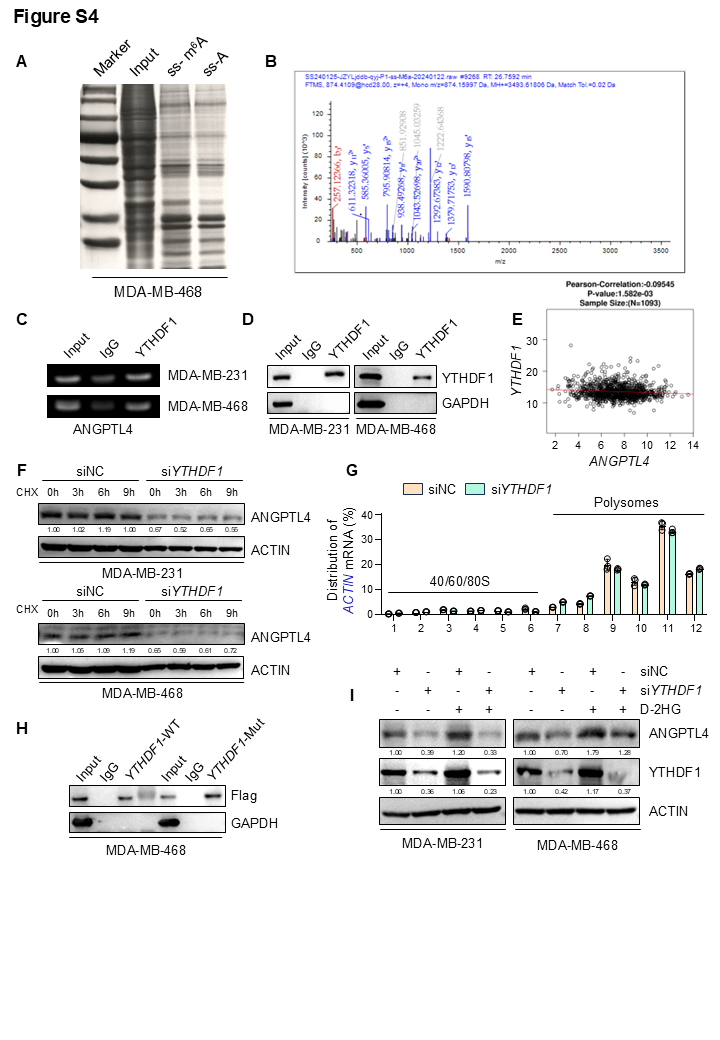


**Figure S4. YTHDF1 regulates the translation of ANGPTL4 in TNBC cells. A.** Silver staining after RNA pull-down assay was performed to identify specific proteins interacted with *ANGPTL4* ss-m^6^A RNA probes. **B.** A peptide spectrum generated by LC–MS/MS revealing YTHDF1 precipitated by *ANGPTL4* ss-m^6^A RNA probes from the lysates of MDA-MB-468 cells. **C.** Representative gel images of YTHDF1-specific RIP-derived RNA analyzed by qPCR. **D.** YTHDF1-specific RIP-derived proteins in MDA-MB-468 cells were measured by western blot. **E.** Correlation between the expression of *YTHDF1* and *ANGPTL4* (Linkedomics database). **F.** Western blot analysis of ANGPTL4 protein expression following 100 μg/ml CHX treatment for indicated time in TNBC cells. **G.** Relative *ACTIN* mRNA distribution in each ribosome fractions in polysome profiling assay was analyzed as positive control by qPCR. **H.** Flag-specific RIP-derived proteins in MDA-MB-468 cells were measured by western blot. **I.** Western blot analysis of protein expression of ANGPTL4 in *YTHDF1* knockdown TNBC cells after D-2HG treatment for 48h. **P* < 0.05, ***P* < 0.01, ****P* < 0.001.


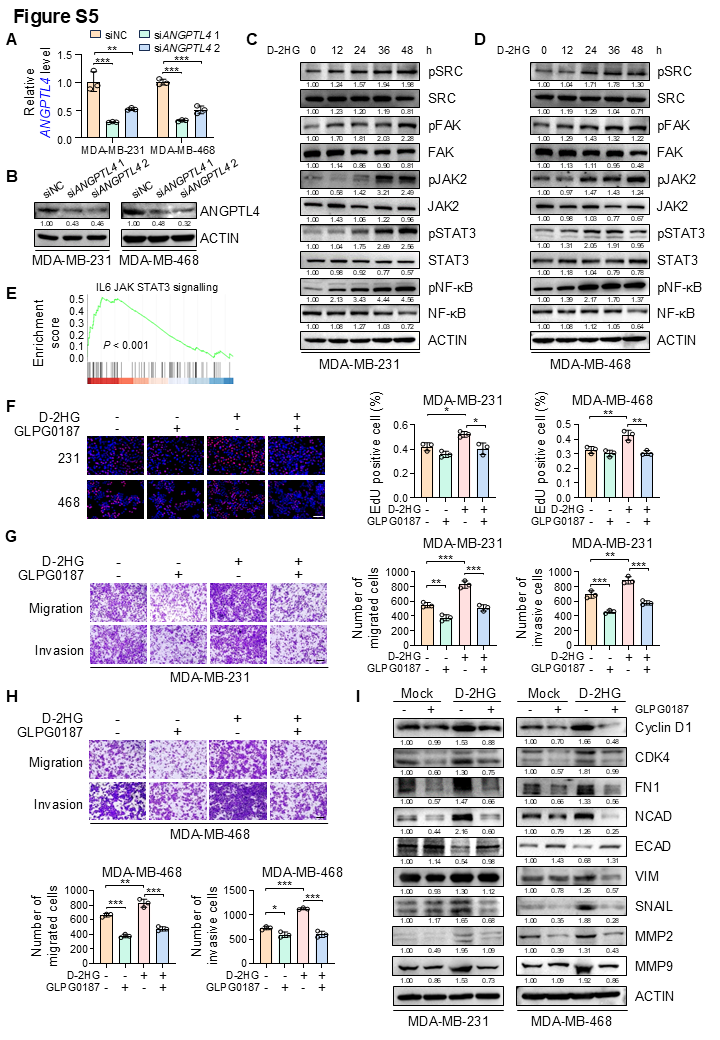
 **Figure S5. Integrin antagonist partially impairs the oncogenic effect of D-2HG in TNBC cells.** **A.** Relative RNA levels of *ANGPTL4* in MDA-MB-231 and MDA-MB-468 cells following *ANGPTL4* knockdown. **B.** Relative protein levels of ANGPTL4 in MDA-MB-231 and MDA-MB-468 cells following *ANGPTL4* knockdown. **C-D.** TNBC cells were treated with 500 μM D-2HG for the indicated duration, and the activation of the integrin/SRC/FAK/JAK2/STAT3 pathway was evaluated by western blot. **E.** GSEA analysis of RNA-seq results showed the enhanced IL6-JAK-STAT3 pathway enrichment in D-2HG-treated MDA-MB-468 cells. **F.** Representative images (left) and quantification (right) of the EdU assay in TNBC cells. Scale bar: 100 μm. **G.** Representative images and quantification of migration and invasion assays in MDA-MB-231 cells. Scale bar: 200 μm. **H.** Same as in G, but in MDA-MB-468 cells. Scale bar: 200 μm. **I.** TNBC cells were pre-treated with 5 ng/mL GLPG0187 for 12h, followed by D-2HG treatment (500 μM) for 48h, western blot showed the protein expression related with cell cycle, migration, and invasion. **P* < 0.05, ***P* < 0.01, ****P* < 0.001.


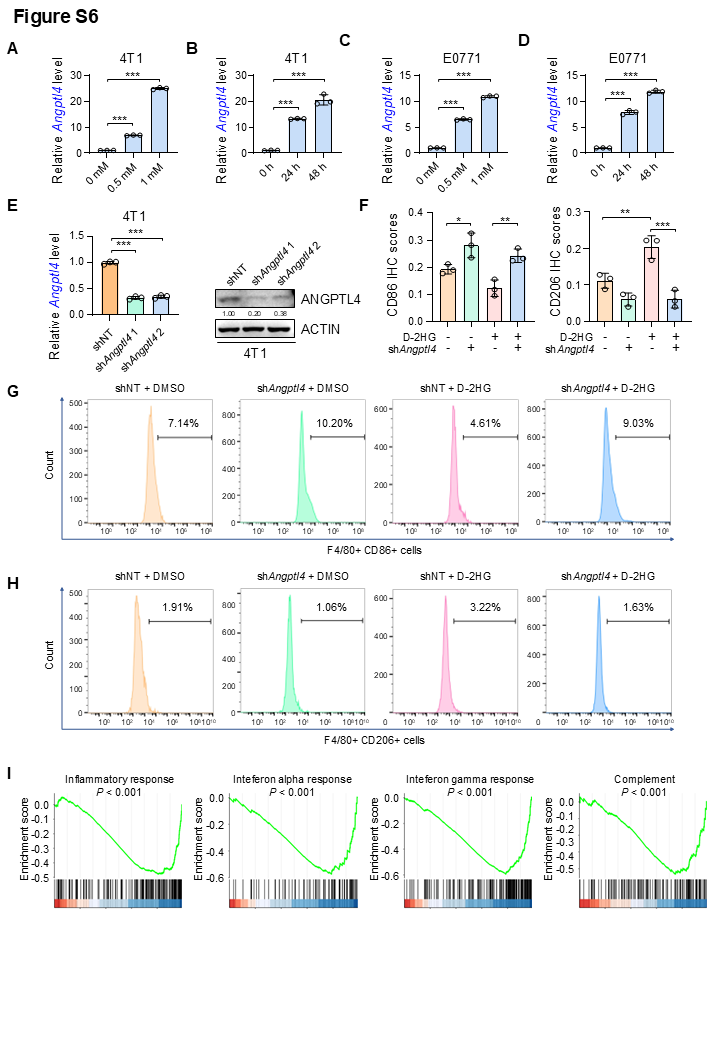


**Figure S6. D-2HG-mediated secretion of ANGPTL4 promotes M2-like** **macrophage polarization in TNBC. A.** qPCR analysis of *Angptl4* in 4T1 cells treated with indicated concentrations of D-2HG for 48 h. **B.** qPCR analysis of *Angptl4* in 4T1 cells treated with D-2HG (500 μM) with indicated time points. **C.** Same as A, but in E0771 cells. **D.** Same as B, but in E0771 cells. **E.** Validation of *Angptl4* knockdown in 4T1 cell line. **F.** Quantification of CD86 and CD206 immunohistochemical staining. **G-H.** Flow cytometry analysis of M1 (F4/80+CD86+) and M2 (F4/80+CD206+) marker expression of tumor-infiltrating macrophages. **I.** GSEA analysis of RNA-seq results showed several downregulated immune-related pathways in E0771 conditioned medium (CM) combined with mANGPTL4 treated peritoneal macrophages. **P* < 0.05, ***P* < 0.01, ****P* < 0.001.
